# Supplementary material for: Comparative fertility and pregnancy outcomes after local treatment for cervical intraepithelial neoplasia and stage 1a1 cervical cancer: protocol for a systematic review and network meta-analysis from the CIRCLE group
Source: BMJ Open. 2019 Oct 21;9(10):e028009. doi: 10.1136/bmjopen-2018-028009 (PMC6803140; doi:10.1136/bmjopen-2018-028009)
Supplement: Author's manuscript [file bmjopen-2018-028009.draft_revisions.pdf]

BMJ Open is committed to open peer review. As part of this commitment we make the peer review history of every article we publish publicly available.

When an article is published we post the peer reviewers' comments and the authors' responses online. We also post the versions of the paper that were used during peer review. These are the versions that the peer review comments apply to.

The versions of the paper that follow are the versions that were submitted during the peer review process. They are not the versions of record or the final published versions. They should not be cited or distributed as the published version of this manuscript.

BMJ Open is an open access journal and the full, final, typeset and author-corrected version of record of the manuscript is available on our site with no access controls, subscription charges or pay-per-view fees (http://bmjopen.bmj.com).

If you have any questions on BMJ Open's open peer review process please email info.bmjopen@bmj.com

BMJ Open

Comparative fertility and pregnancy outcomes after local treatment for cervical intra-epithelial neoplasia and stage 1a1 cervical cancer: protocol for a systematic review and network meta-analysis from the CIRCLE Group

Journal:	*BMJ Open*
Manuscript ID	bmjopen-2018-028009
Article Type:	Protocol
Date Submitted by the Author:	05-Dec-2018
Complete List of Authors:	Athanasiou, Antonios; Institute of Reproductive and Developmental Biology, Department of Surgery & Cancer, Faculty of Medicine, Imperial College London; Imperial College Healthcare NHS Trust<br>Veroniki, Areti Angeliki; Institute of Reproductive and Developmental Biology, Department of Surgery & Cancer, Faculty of Medicine, Imperial College London; Panepistimio Ioanninon, School of Education, Department of Primary Education<br>Efthimiou, Orestis; University of Bern, Institute of Social and Preventive Medicine (ISPM)<br>Kalliala, Ilkka; Institute of Reproductive and Developmental Biology, Department of Surgery & Cancer, Faculty of Medicine, Imperial College London; University of Helsinki and Helsinki University Hospital, Department of Obstetrics and Gynaecology<br>Naci, Huseyin; London School of Economics, Department of Health Policy<br>Lever, Sarah; Institute of Reproductive and Developmental Biology, Department of Surgery & Cancer, Faculty of Medicine, Imperial College London; Imperial College Healthcare NHS Trust<br>Paraskevaidi, Maria; Institute of Reproductive and Developmental Biology, Department of Surgery & Cancer, Faculty of Medicine, Imperial College London<br>Martin-Hirsch, Pierre; Lancashire Teaching Hospitals NHS Foundation Trust, Department of Gynaecologic Oncology<br>Bennett, Philip; Institute of Reproductive and Developmental Biology, Department of Surgery & Cancer, Faculty of Medicine, Imperial College London; Imperial College Healthcare NHS Trust<br>Paraskevaidis, Evangelos; University of Ioannina and University Hospital of Ioannina, Department of Obstetrics & Gynaecology; Imperial College Healthcare NHS Trust<br>Salanti, Georgia; University of Bern, Institute of Social and Preventive Medicine (ISPM)<br>Kyrgiou, Maria; Institute of Reproductive and Developmental Biology, Department of Surgery and Cancer, Faculty of Medicine, Imperial College London; Imperial College Healthcare NHS Trust
Keywords:	"Cervical Intraepithelial Neoplasia/surgery"[Mesh], "Cervical Intraepithelial Neoplasia/therapy"[Mesh], "Conization/adverse effects"[Mesh], "Ablation Techniques/adverse effects"[Mesh], "Pregnancy/complications"[Mesh], "Premature Birth"[Mesh

SCHOLARONE™
Manuscripts

Comparative fertility and pregnancy outcomes after local treatment for cervical intra-epithelial neoplasia and stage 1a1 cervical cancer: protocol for a systematic review and network meta-analysis from the CIRCLE Group

Antonios Athanasiou[1,2], Areti Angeliki Veroniki[1,3], Orestis Efthimiou[4], Ilkka Kalliala[1,5], Huseyin Naci[6], Sarah Lever[1,2], Maria Paraskevaidi[1], Pierre Martin-Hirsch[7], Phillip Bennett[1,2], Evangelos Paraskevaidis[2,8], Georgia Salanti[4], Maria Kyrgiou[1,2]

[1]Institute of Reproductive and Developmental Biology, Department of Surgery & Cancer, Faculty of Medicine, Imperial College London, UK
[2]Imperial Healthcare NHS Trust, London, UK
[3]Department of Primary Education, School of Education, University of Ioannina, Greece
[4]Institute of Social and Preventive Medicine (ISPM), University of Bern, Finkenhubelweg 11, 3012 Bern, Switzerland
[5]Department of Obstetrics and Gynaecology, University of Helsinki and Helsinki University Hospital, Finland
[6]Department of Health Policy, London School of Economics & Political Science, UK
[7]Department of Gynaecologic Oncology, Lancashire Teaching Hospitals, Preston, UK
[8]Department of Obstetrics & Gynaecology, University of Ioannina and University Hospital of Ioannina, Greece

AA: a.athanasiou@imperial.ac.uk; AAV: a.veroniki@imperial.ac.uk; OE: oremiou@gmail.com; IK: i.kalliala@imperial.ac.uk; HN: h.naci@lse.ac.uk; SL: s.lever@imperial.ac.uk; MP: m.paraskevaidi@imperial.ac.uk; PMH: pierre.martin-hirsch@lthtr.nhs.uk; PB: p.bennett@imperial.ac.uk; EP: eparaske@cc.uoi.gr; GS: georgia.salanti@ispm.unibe.ch; MK: m.kyrgiou@imperial.ac.uk

Corresponding author:
Maria Kyrgiou, MSc, PhD, MRCOG
3rd Floor, IRDB, Department of Surgery & Cancer
Imperial College London, Hammersmith Campus
Du Cane Road, W12 0NN, London
Email: m.kyrgiou@imperial.ac.uk - Tel: +44 2075942177

Word Count: 3373

For peer review only

For peer review only - http://bmjopen.bmj.com/site/about/guidelines.xhtml

ABSTRACT

Introduction: There are several local treatment methods for cervical intra-epithelial neoplasia that remove or ablate a cone-shaped part of the uterine cervix. There is evidence to suggest that these increase the risk of preterm birth and that this is higher for techniques that remove larger parts of the cervix, although the data is conflicting. We present a protocol for a systematic review and network meta-analysis that will update the evidence and compare all treatments in terms of fertility and pregnancy complications.

Methods and Analysis: We will search electronic databases to identify randomised controlled trials (RCTs) and cohort studies comparing the fertility and pregnancy outcomes amongst different excisional and ablative treatment techniques and/or to untreated controls. The primary outcome will be preterm birth (PTB; <37weeks). Secondary outcomes will include severe or extreme PTB, prelabour rupture of membranes, low birth weight (<2500gr), neonatal intensive care unit admission, perinatal mortality, total pregnancy rates, 1st and 2nd trimester miscarriage. We will search for published and unpublished studies in electronic databases, trial registries and we will hand-search references of published papers. We will assess the risk of bias in RCTs and cohort studies using tools developed by the Cochrane Collaboration. Two investigators will independently assess the eligibility, abstract the data and assess the risk of bias of the identified studies. For each outcome, we will perform a meta-analysis for each treatment comparison and a network meta-analysis once the transitivity assumption holds, using the odds ratio for dichotomous data. We will use CINEMA to assess the quality of the evidence for the primary outcome.

Ethics and dissemination: Ethical approval is not required. Results will be disseminated to academic beneficiaries, medical practitioners, patients and the public.

PROSPERO registration number: CRD42018115495

ARTICLE SUMMARY

Strengths and limitations of this study

- This will be the first network meta-analysis to produce comprehensive summaries of the relative reproductive morbidity of treatment methods for cervical pre-invasive and early invasive disease.
- The results will be used to inform effective patient counselling at colposcopy and antenatal clinics and will contribute to the development of clinical guidelines.
- One possible limitation of this review is that we expect to find mainly retrospective cohort studies at high risk of recall, selection and publication bias.

KEYWORDS

"Cervical Intraepithelial Neoplasia/surgery"[Mesh]
"Cervical Intraepithelial Neoplasia/therapy"[Mesh]
"Conization/adverse effects"[Mesh]
"Ablation Techniques/adverse effects"[Mesh]
"Pregnancy/complications"[Mesh]

For peer review only - http://bmjopen.bmj.com/site/about/guidelines.xhtml

"Premature Birth"[Mesh]

INTRODUCTION

The introduction of systematic call and recall screening programmes in the UK has resulted in a profound decrease in the incidence and mortality from cervical cancer, as pre-invasive precursors (cervical intra-epithelial neoplasia; CIN) can be detected by the screening programme and treated[1]. In England alone, 3.6 million women aged 25 to 64 attended for screening in 2013-14 and over 23,800 treatment procedures were carried out[2].

Local conservative treatment for cervical pre-invasive and early invasive disease removes or ablates a cone-shaped part of the cervix containing the precancerous cells. The choice of technique varies within the UK, across Europe and beyond. In some countries, knife excision (cold knife conisation; CKC) is still regularly performed; in others, ablation (LA) or conisation (LC) with the laser beam is common practice. In the UK, large loop excision of the transformation zone (LLETZ) is the preferred treatment, with some units offering alternative techniques more frequently than others. This preference is because LLETZ is quick, easy to do and of low cost.

The mean age of women undergoing CIN treatment is similar to the age of women having their first child. Although, previously, complications from treatment were thought to be relatively mild and uncommon, an increasing body of retrospective observational studies and meta-analyses suggested that treatment, particularly excision, adversely affects future reproduction and the risk of prematurity[3-8]. It has been suggested that the frequency and severity of the observed adverse events is higher for the more radical techniques and with increasing cone depth[4 7 9-14]. Preterm birth (PTB) is a major cause of neonatal death and disability and represents an enormous cost to the health services and the society in general.

Although all treatment techniques are highly effective in preventing recurrent pre-cancerous disease and future invasion[15], some of the data on the risk of reproductive morbidity for the treatment methods has been conflicting[3 5 7 10 16-18] and their comparative reproductive morbidity remains unclear. With some authors raising concerns that the progressive reduction in the radicality of treatment has led to increased risk of future post-treatment invasive disease[19 20], and others advocating the move to less radical techniques, such as laser ablation, for the prevention of treatment-associated adverse obstetric outcomes, such as preterm birth or perinatal mortality[4 21], high-quality synthesis of the current evidence base is an urgent unmet need. Given the premalignant nature of the condition, an RCT comparing the various treatment technique to no treatment will never be conducted.

The quantification of the comparative reproductive morbidity of different treatment techniques and cone lengths has become a women's health priority. This requires high-quality synthesis of the evidence in comprehensive summaries that will become available for effective patient counselling at colposcopy and antenatal clinics for patients, clinicians and policy makers. A clinical ranking of treatments with regards to the risk of preterm birth may

For peer review only - http://bmjopen.bmj.com/site/about/guidelines.xhtml

allow the quantification of risk and the detection of women at high risk of PTB that would benefit from intensive surveillance antenatally, while minimising the unnecessary interventions for those at lower risk.

The key methodological vehicle to synthesize evidence is systematic reviews and their quantitative component, meta-analysis. Network meta-analysis (NMA) is an extension of pairwise meta-analysis, which can be used to estimate the relative effectiveness of several competing treatments. An NMA has never been used before to assess the comparative efficacy and harms of the different treatment techniques in this field. For every treatment comparison NMA synthesizes both direct evidence (i.e. coming from studies comparing head-to-head the treatments of interest) and indirect evidence (i.e. coming from studies comparing the treatments of interest via an intermediate common comparator)[22-25]. In addition, NMA allows the estimation of relative effects between all available treatments, can lead to an increased precision as compared to the pairwise meta-analysis and provides a ranking of the available competing treatments. The potential of NMA has been recognized by the National Institute of Clinical Excellence (NICE)[26] and several international Health Technology Assessment (HTA) agencies[27 28].

The objective of this systematic review and NMA is to update the evidence and to compare the various local treatment methods to manage CIN in terms of fertility, early (<24 weeks of gestation) and late (>24 weeks) pregnancy complications. This is part of the CIRCLE project (**C**ervical Cancer **I**ncidence, C**I**N **R**ecurrence and **R**eproduction after **L**ocal **E**xcision), which aims to generate a clinically useful raking of alternative options for treatment of CIN according to their efficacy (risk of pre-invasive and invasive recurrence), morbidity and cost-effectiveness.

METHODS AND ANALYSIS

This protocol complies with the Preferred Reporting Items for Systematic Review and Meta-Analysis Protocols (PRISMA-P) (Supplementary File 1)[29]. Any changes in this protocol will be recorded in an updated version of the PROSPERO registration.

Criteria for considering studies for this review

Types of participants

We will include women of all ages with a prior history of local surgical treatment for CIN or micro-invasive early cervical cancer (stage IA1). Their status can be confirmed with histological or cytological diagnosis and irrespective of the grade of the treated lesion for both squamous and glandular intra-epithelial neoplasia. Studies recruiting solely women at high risk of preterm birth (such as previous history of preterm birth) or studies including only patients treated during pregnancy will be excluded.

Types of interventions

We aim to compare 9 different excisional or ablative techniques used for conservative treatment for cervical intraepithelial neoplasia (CIN). The excisional techniques include cold

For peer review only - http://bmjopen.bmj.com/site/about/guidelines.xhtml

knife conisation (CKC); laser conisation (LC); needle excision of the transformation zone (NETZ), also known as straight wire excision of the transformation zone (SWETZ); large loop excision of the transformation zone (LLETZ), also known as loop electrosurgical excisional procedure (LEEP); Fischer cone biopsy excision (FCBE). The ablative techniques include radical point diathermy (RD); cryotherapy (CT); cold coagulation (CC); laser ablation (LA). We will include studies comparing any of these treatments with each other or with no treatment. Figure 1 shows a network example of all possible comparisons between eligible interventions for the primary analysis. If the specific treatment technique is not specified, these will be grouped under the broader categories excision (ENS) or ablation (ANS).

Outcome measures

Primary outcome

- PTB, defined as less than 37 weeks of gestation

Secondary outcomes

- Severe PTB, defined as less than 32/34 weeks of gestation
- Extreme PTB, defined as less than 28/30 weeks of gestation
- PROM (Prelabour Rupture of Membranes) defined as membrane rupture before the onset of labour
- Low Birth Weight (LBW), defined as infant born weighing less than 2500g (late neonatal obstetric outcome)
- Neonatal Intensive Care Unit (NICU) admission
- Perinatal mortality, defined as the number of stillbirths and neonatal deaths occurring within 28 days after birth
- Total pregnancy rate
- 1st trimester miscarriage, defined as miscarriage at less than 12 weeks of gestation
- 2nd trimester miscarriage, defined as miscarriage between 12-24 weeks of gestation

Outcomes and their classification into primary or secondary were decided after clinical experts' opinion.

Types of studies

We will include randomised controlled trials (RCTs), quasi-RCTs and cohort studies comparing fertility and early (<24 weeks of gestation) or late (>24 weeks of gestation) pregnancy outcomes amongst surgical techniques and those that compare to no treatment. Studies that do not perform a comparison between treatments (i.e. "single-arm studies") will be excluded. Studies that compared a treatment with an untreated group will be included irrespective of the type of the untreated group (e.g. studies that used data from untreated women from the general population; studies that used self-matching, i.e. including women with pregnancies before and after treatment; studies that used data from women with a history of abnormal cytology/HPV infection/untreated CIN). There will be no time or language restriction.

For peer review only - http://bmjopen.bmj.com/site/about/guidelines.xhtml

For peer review only

Information sources and search strategy

An experienced librarian will search The Cochrane Gynaecological Cancer Specialised Register; Cochrane Central Register of Controlled Trials (CENTRAL); MEDLINE; and EMBASE for eligible studies from inception. The search algorithms for these databases are presented in Supplementary File 2. We will search Metaregister, Physicians Data Query, www.controlled-trials.com/rct, www.clinicaltrials.gov and www.cancer.gov/clinicaltrials for ongoing studies. There will be no time or language restriction.

We will contact the corresponding author of any relevant ongoing trials for further information and unpublished data. In an attempt to identify any articles missed by the initial search, we will use the 'related articles' feature in MEDLINE. We will also hand search the references of the retrieved articles and meta-analyses. We will search conference proceedings and abstracts through ZETOC (http://zetoc.mimas.ac.uk), and theses through WorldCat Dissertations. The selected conferences will include: British Society of Colposcopy and Cervical Pathology; International Federation of Cervical Pathology and Colposcopy; Annual Meeting of European Federation of Colposcopy; Annual Meeting of the American Society of Colposcopy and Cervical Pathology. We will contact experts in the field, including directors of UK cancer and colposcopy registries, to identify further reports of studies. We will include both published and unpublished studies.

Study selection

We will download abstracts retrieved into a reference management software, Zotero. Then, two persons will independently review titles and abstracts retrieved by the search (level 1). At level 2, we will obtain the full text of all included articles and two reviewers will independently use the same inclusion criteria to determine eligibility. Disagreements at any level will be resolved via discussion with a third member of the review team.

Data collection

Two reviewers will extract data independently using a standardised data collection form in Excel. Disagreements will be resolved through discussion. Information extracted will include study characteristics (such as author, publication year and study design), participants (such as age, CIN grade and smoking) and comparison group characteristics, setting, inclusion/exclusion criteria, intervention details, outcome measures and dropout rates. In RCTs we will prefer arm-level data (number of events and sample size per intervention arm), but if these are missing, the study-level data will be used in the analysis, e.g. reported odds ratios (ORs) and a measure of their uncertainty (e.g. confidence interval). In observational studies we will extract estimates of treatment effects that are adjusted for the lack of randomisation, i.e. after taking into account the impact of potential confounders, or, if these are missing, the reported unadjusted estimates, as well as the corresponding uncertainty measure.

Risk of bias assessment

For peer review only

Results of the meta-analyses will be interpreted in light of risk of bias assessment of the included studies. Two investigators will independently assess the methodological quality/risk of bias of the studies that fulfil the eligibility criteria and differences will be resolved by discussion with a third investigator.

For RCTs, the risk of bias will be assessed using the tool[30] developed by Cochrane assessing the following domains: randomisation process, deviations from the intended interventions, missing outcome data, measurement of the outcome and selection of the reported result. The risk of bias in each domain, as well as the overall risk of bias, will be rated as "low risk", "some concerns", or "high risk", after answering the signalling questions of each domain with "Yes", "Potentially Yes", "Potentially No" or "No". When inadequate details are reported in the study to be able to rate a risk of bias item, we will contact the study authors for additional information.

For non-randomised studies (NRS), we will use the ROBINS-I tool[31] developed by the Cochrane collaboration that facilitates the evaluation of the risk of bias by considering that each NRS is an attempt to mimic an RCT comparing the effects of the intervention or exposure studied. During the review stage, we will evaluate the risk of bias in the following domains: confounding, selection of participants into the study, classification of interventions, deviations from intended interventions, missing data, measurement of outcomes and selection of the reported results. The confounding factors that we will evaluate are age, parity and smoking. Each ROBINS-I domain and the overall risk of bias will be assessed as "low", "moderate", "serious", or "critical", after answering the signalling questions of each domain with "Yes", "Potentially Yes", "Potentially No" or "No".

Statistical synthesis

Characteristics of included studies and Network

We will generate descriptive statistics for eligible studies and study population characteristics, describing the types of comparisons and important clinical or methodological variables (such as publication year, study design and source of data). We will present the evidence in a network diagram per outcome. The total number of patients will be reflected in the size of the nodes, while the weight of each edge will be proportional to the number of studies per treatment comparison.

Pairwise meta-analyses

We will synthesise data to obtain summary ORs for dichotomous outcomes with a 95% CI in an inverse variance random-effects model assuming that the studies are estimating different but related treatment effects. In each meta-analysis, we will estimate the between-study variance with the restricted maximum likelihood estimator[32 33] and its 95% CI using the Q-profile approach[34]. We will assess between-study variance using the I^2 statistic along a 95% confidence interval (CI)[35 36]. We will estimate each summary effect size and its 95% CI using the Hartung-Knapp-Sidik-Jonkman method[32 37 38] to handle meta-analyses that include a small number of studies. All meta-analyses will be conducted in R[39] using the *metafor* package[40].

For peer review only - http://bmjopen.bmj.com/site/about/guidelines.xhtml

Network meta-analyses (NMA)

Data synthesis

We will fit a random effects NMA model, taking into account the correlation induced by multi-arm studies[41]. We will use a random-effects model, since we anticipate methodological and clinical heterogeneity across studies. We will assume a common between-study variance parameter for all comparisons in the network, so that treatment comparisons informed by a single study can borrow strength from the remaining network[42 43]. Clinically, this assumption is reasonable because all treatments included in the network are of the same nature. We will estimate the common between-study variance with the DerSimonian and Laird method of moments approach[44].

We expect that we will include several NRS in our dataset. In that case, we will employ the methods described by Efthimiou et al.[45], i.e. a "design-adjusted", and a three-level hierarchical NMA model. Using these methods, we will incorporate the totality of available information, both randomized and observational, in a joint NMA. We will start by analysing the study-specific estimates from the NRS at face value. Then, in extensive sensitivity analyses we will explore the impact of assigning different levels of credibility and subsequently down-weighting the NRS, according to experts' opinion and the risk of bias as assessed in ROBINS-I.

For each treatment comparison we will report the estimated OR, the 95% CI and the 95% prediction intervals. We will also estimate the ranking probabilities for all treatments of being at each possible rank for each intervention. Thus, we will obtain a treatment hierarchy using the surface under the cumulative ranking curve (SUCRA) or P-scores and mean ranks[46]. A rank-heat plot will be used to depict the SUCRA values or P-scores for all outcomes[47 48]. All NMAs will be fit in R[39] with the *netmeta*[49] and *rjags*[50] package.

We will repeat our NMAs after grouping all excisional and all ablative techniques together; see groupings of treatments in Figure 1.

Assessment of the transitivity assumption

NMA rests on the assumption of transitivity, i.e. that effect modifiers have a similar distribution across treatment comparisons in a network[24 51 52]. In order to assess the plausibility of this assumption, we will summarize study and patient-level characteristics that are expected to influence relative treatment effects, for each pairwise comparison for which direct evidence is available in the network. In this NMA, the most important effect modifiers are expected to be year of study, method of ascertainment of exposure/outcome (hospital records, registries or interviews/questionnaires), age, parity, smoking and CIN grade. We will visually inspect the similarity of the identified studies in terms of these effect modifiers. We will investigate the inclusion and exclusion criteria of all studies, to make sure that patients, treatments and outcomes in the studies are sufficiently similar in all aspects that are expected to modify relative treatment effects.

For peer review only

Assessment of statistical inconsistency

Checking the network for inconsistency offers an additional way of assessing the validity of the transitivity assumption. In order to evaluate the presence of inconsistency locally, we will separate the indirect from the direct evidence for each comparison and infer about their differences following the back-calculation method[53]. We will also follow a global approach for assessing consistency in the network, by applying the design-by-treatment interaction model[54]. Simulations suggest that inconsistency tests have low power to detect true inconsistency[55 56]. Therefore, we will conceptually assess the transitivity assumption (see previous paragraph) even in the absence of evidence for inconsistency. We will perform both local (back-calculation method) and global (design-by-treatment interaction model) assessments in R[39] using the *netmeta* package[49].

When a network includes both randomised and non-randomised evidence, we will also explore differences between the different types of evidence, as discussed in Efthimiou et al[45]. For each treatment comparison there may be up to four different types of evidence: direct randomised, indirect randomised, direct non-randomised and indirect non-randomised. We will summarise all evidence by type, for each treatment comparison. If data permits, important discrepancies between these types will be further investigated, as they might indicate a breach of the transitivity assumption (e.g. when randomised and non-randomised evidence are very different in terms of populations, interventions, etc.), or the presence of important, unaccounted confounding in the non-randomised evidence. If a source of disagreement is identified, it will be included in our analysis through network meta-regression models[23].

Exploring heterogeneity and inconsistency: subgroup analyses, meta-regression, and sensitivity analyses

We will assess the extent of statistical heterogeneity by inspecting the 95% prediction intervals and by comparing the estimated value of the between-study variance with the empirical distribution derived by Turner et al for dichotomous data[57]. For the primary outcome, we will explore the following possible sources of heterogeneity and inconsistency: year of study, method of ascertainment of exposure/outcome (hospital records, registries or interviews/questionnaires), age, parity, smoking and CIN grade. If sufficient studies are available, the role of these variables will be explored by means of subgroup analyses (categorical characteristics) or network meta-regressions (continuous characteristics).

Reporting bias and small-study effects

In order to assess possible existence of small studies giving different effect estimates than larger studies, we will visually explore the funnel-plots for each treatment against the untreated group (using the relevant studies) when at least 10 studies inform the underling treatment comparison. We will also assess for small-study effects using the comparison adjusted funnel plot[58] and will conduct a network meta-regression using the study variance as a covariate[59 60].

Assessment of the credibility of the evidence

For peer review only - http://bmjopen.bmj.com/site/about/guidelines.xhtml

We will evaluate the credibility of the evidence contributing to the network estimates in the primary outcome using CINEMA[61] (http://cinema.ispm.ch/). Two team members will determine the degree of confidence in the estimated NMA results by assessing the six CINEMA domains: within-study bias (i.e., risk of bias in the included studies), across-study bias (i.e., publication and reporting bias), indirectness, imprecision, heterogeneity and incoherence (i.e., differences between direct and indirect evidence)[61]. Each network summary estimate will initially be judged as high quality, but it will be downgraded if this is judged appropriate according to the six domains. Judgements within each domain will be summarized for each NMA relative treatment effect as: very low, low, moderate or high.

Patient and Public involvement

We have discussed the project with Jo's Cervical Cancer Trust (a UK charity who supports patients affected by cervical pre-invasive or invasive cervical disease and campaigns for excellence in cervical cancer treatment and prevention).

Two patient representatives through Jo's Trust with personal experience of cervical disease have assisted us in the design of the study and the development of research questions. We aim to recruit more patients through Jo's Voice, who will help us understand the key priorities from patients' perspective, produce lay summaries and disseminate the results to the wider public.

ETHICS AND DISSEMINATION

This review does not require ethical approval. We identified four groups of potential stakeholders (academic beneficiaries; health-related agencies and decision-makers; medical practitioners; patients and public) and specific action items to effectively target them. We will publish papers in influential open access journals and we will present data at high-profile conferences. We will make the datasets available to the wider research community. We will organise a workshop with key stakeholders. We will develop information sheets and briefings, highlighting the key findings and circulate newsletters. We will engage the press with presentations and social media interviews and we will work closely with Jo's Trust charity that plays an important role in educating patient communities.

Figure 1. Network of possible pairwise comparisons between eligible treatment methods
Abbreviations: **FCBE**=Fischer cone biopsy excision; **LLETZ**= large loop excision of the transformation zone, also known as **LEEP**=loop electrosurgical excisional procedure; **NETZ**=needle excision of the transformation, also known as **SWETZ**=straight wire excision of the transformation zone; **LC**=laser conisation; **CKC**=cold knife conisation; **LA**=laser ablation; **CC**=cold coagulation; **CT**=cryotherapy; **RD**=radical point diathermy

REFERENCES

1. Quinn M, Babb P, Jones J, et al. Effect of screening on incidence of and mortality from cancer of cervix in England: evaluation based on routinely collected statistics. *BMJ* 1999;318(7188):904-8.

For peer review only - http://bmjopen.bmj.com/site/about/guidelines.xhtml

2. PublicHealthEngland. Cervical screening: programme overview. http://www.cancerscreening.nhs.uk/cervical, 2015.
3. Kyrgiou M, Koliopoulos G, Martin-Hirsch P, et al. Obstetric outcomes after conservative treatment for intraepithelial or early invasive cervical lesions: systematic review and meta-analysis. *Lancet* 2006;367(9509):489-98. doi: 10.1016/s0140-6736(06)68181-6 [published Online First: 2006/02/14]
4. Arbyn M, Kyrgiou M, Simoens C, et al. Perinatal mortality and other severe adverse pregnancy outcomes associated with treatment of cervical intraepithelial neoplasia: meta-analysis. *BMJ* 2008;337:a1284. doi: 10.1136/bmj.a1284 [published Online First: 2008/09/20]
5. Kyrgiou M, Mitra A, Arbyn M, et al. Fertility and early pregnancy outcomes after treatment for cervical intraepithelial neoplasia: systematic review and meta-analysis. *Bmj* 2014;349:g6192. doi: 10.1136/bmj.g6192 [published Online First: 2014/10/30]
6. Kyrgiou M, Mitra A, Arbyn M, et al. Fertility and early pregnancy outcomes after conservative treatment for cervical intraepithelial neoplasia. *Cochrane Database Syst Rev* 2015(9):Cd008478. doi: 10.1002/14651858.CD008478.pub2 [published Online First: 2015/09/30]
7. Kyrgiou M, Athanasiou A, Paraskevaidi M, et al. Adverse obstetric outcomes after local treatment for cervical preinvasive and early invasive disease according to cone depth: systematic review and meta-analysis. *Bmj* 2016;354:i3633. doi: 10.1136/bmj.i3633 [published Online First: 2016/07/30]
8. Kyrgiou M, Athanasiou A, Kalliala IEJ, et al. Obstetric outcomes after conservative treatment for cervical intraepithelial lesions and early invasive disease. *Cochrane Database Syst Rev* 2017;11:Cd012847. doi: 10.1002/14651858.Cd012847 [published Online First: 2017/11/03]
9. Kyrgiou M, Arbyn M, Martin-Hirsch P, et al. Increased risk of preterm birth after treatment for CIN. *Bmj* 2012;345:e5847. doi: 10.1136/bmj.e5847 [published Online First: 2012/09/07]
10. Castanon A, Landy R, Brocklehurst P, et al. Risk of preterm delivery with increasing depth of excision for cervical intraepithelial neoplasia in England: nested case-control study. *BMJ* 2014;349:g6223. [published Online First: 2014/11/05]
11. Noehr B, Jensen A, Frederiksen K, et al. Depth of cervical cone removed by loop electrosurgical excision procedure and subsequent risk of spontaneous preterm delivery. *Obstet Gynecol* 2009;114(6):1232-8. doi: 10.1097/AOG.0b013e3181bf1ef2 [published Online First: 2009/11/26]
12. Khalid S, Dimitriou E, Conroy R, et al. The thickness and volume of LLETZ specimens can predict the relative risk of pregnancy-related morbidity. *Bjog* 2012;119(6):685-91. doi: 10.1111/j.1471-0528.2011.03252.x [published Online First: 2012/02/15]
13. Founta C, Arbyn M, Valasoulis G, et al. Proportion of excision and cervical healing after large loop excision of the transformation zone for cervical intraepithelial neoplasia. *Bjog* 2010;117(12):1468-74. doi: 10.1111/j.1471-0528.2010.02709.x [published Online First: 2010/09/16]
14. Kyrgiou M, Valasoulis G, Stasinou SM, et al. Proportion of cervical excision for cervical intraepithelial neoplasia as a predictor of pregnancy outcomes. *Int J Gynaecol Obstet*

For peer review only

2015;128(2):141-7. doi: 10.1016/j.ijgo.2014.07.038 [published Online First: 2014/12/03]

15. Martin-Hirsch PP, Paraskevaidis E, Bryant A, et al. Surgery for cervical intraepithelial neoplasia. *Cochrane Database Syst Rev* 2013(12):Cd001318. doi: 10.1002/14651858.CD001318.pub3 [published Online First: 2013/12/05]
16. Conner SN, Frey HA, Cahill AG, et al. Loop electrosurgical excision procedure and risk of preterm birth: a systematic review and meta-analysis. *Obstet Gynecol* 2014;123(4):752-61. doi: 10.1097/AOG.0000000000000174 [published Online First: 2014/05/03]
17. Danhof NA, Kamphuis EI, Limpens J, et al. The risk of preterm birth of treated versus untreated cervical intraepithelial neoplasia (CIN): a systematic review and meta-analysis. *Eur J Obstet Gynecol Reprod Biol* 2015;188:24-33. doi: 10.1016/j.ejogrb.2015.02.033 [published Online First: 2015/03/17]
18. Jin G, LanLan Z, Li C, et al. Pregnancy outcome following loop electrosurgical excision procedure (LEEP) a systematic review and meta-analysis. *Arch Gynecol Obstet* 2014;289(1):85-99. doi: 10.1007/s00404-013-2955-0 [published Online First: 2013/07/12]
19. Strander B, Hällgren J, Sparén P. Effect of ageing on cervical or vaginal cancer in Swedish women previously treated for cervical intraepithelial neoplasia grade 3: population based cohort study of long term incidence and mortality. *BMJ* 2014;348:f7361. [published Online First: 2014/01/14]
20. Arbyn M, Kyrgiou M, Gondry J, et al. Long term outcomes for women treated for cervical precancer. *Bmj* 2014;348:f7700. doi: 10.1136/bmj.f7700 [published Online First: 2014/01/16]
21. Paraskevaidis E, Kyrgiou M, Martin-Hirsch P. Have we dismissed ablative treatment too soon in colposcopy practice? *Bjog* 2007;114(1):3-4. doi: 10.1111/j.1471-0528.2006.01178.x [published Online First: 2007/01/20]
22. Caldwell DM, Ades AE, Higgins JP. Simultaneous comparison of multiple treatments: combining direct and indirect evidence. *BMJ* 2005;331(7521):897-900. doi: 10.1136/bmj.331.7521.897
23. Salanti G, Marinho V, Higgins JP. A case study of multiple-treatments meta-analysis demonstrates that covariates should be considered. *J Clin Epidemiol* 2009;62(8):857-64. doi: 10.1016/j.jclinepi.2008.10.001 [published Online First: 2009/01/20]
24. Cipriani A, Higgins JP, Geddes JR, et al. Conceptual and technical challenges in network meta-analysis. *Ann Intern Med* 2013;159(2):130-7. doi: 10.7326/0003-4819-159-2-201307160-00008
25. Efthimiou O, Debray TP, van Valkenhoef G, et al. GetReal in network meta-analysis: a review of the methodology. *Res Synth Methods* 2016;7(3):236-63. doi: 10.1002/jrsm.1195 [published Online First: 2016/01/13]
26. NICE. Developing NICE guidelines: the manual. Process and methods (PMG20). Reviewing research evidence 2017 [Available from: https://www.nice.org.uk/process/pmg20/chapter/reviewing-research-evidence.

For peer review only - http://bmjopen.bmj.com/site/about/guidelines.xhtml

27. Caldwell DM, Dias S, Welton NJ. Extending Treatment Networks in Health Technology Assessment: How Far Should We Go? *Value Health* 2015;18(5):673-81. doi: 10.1016/j.jval.2015.03.1792 [published Online First: 2015/08/25]
28. CADTH. Network Meta-Analysis 2015 [Available from: https://www.cadth.ca/network-meta-analysis.
29. Moher D, Shamseer L, Clarke M, et al. Preferred reporting items for systematic review and meta-analysis protocols (PRISMA-P) 2015 statement. *Syst Rev* 2015;4:1. doi: 10.1186/2046-4053-4-1 [published Online First: 2015/01/03]
30. Higgins JPT, Sterne JAC, Savović J, et al. A revised tool for assessing risk of bias in randomized trials. In: Chandler J, McKenzie J, Boutron I, et al., eds.: Cochrane Methods. Cochrane Database of Systematic Reviews 2016.
31. Sterne JA, Hernan MA, Reeves BC, et al. ROBINS-I: a tool for assessing risk of bias in non-randomised studies of interventions. *BMJ* 2016;355:i4919. doi: 10.1136/bmj.i4919 [published Online First: 2016/10/14]
32. Veroniki AA, Jackson D, Viechtbauer W, et al. Methods to estimate the between-study variance and its uncertainty in meta-analysis. *Res Synth Methods* 2016;7(1):55-79. doi: 10.1002/jrsm.1164 [published Online First: 2015/09/04]
33. Langan D, Higgins JPT, Jackson D, et al. A comparison of heterogeneity variance estimators in simulated random-effects meta-analyses. *Res Synth Methods* 2018 doi: 10.1002/jrsm.1316 [published Online First: 2018/08/02]
34. Viechtbauer W. Confidence intervals for the amount of heterogeneity in meta-analysis. *Stat Med* 2007;26(1):37-52. doi: 10.1002/sim.2514 [published Online First: 2006/02/08]
35. Higgins JP, Thompson SG, Deeks JJ, et al. Measuring inconsistency in meta-analyses. *BMJ* 2003;327(7414):557-60. doi: 10.1136/bmj.327.7414.557 [published Online First: 2003/09/06]
36. Veroniki AA, Jackson D, Viechtbauer W, et al. Recommendations for quantifying the uncertainty in the summary intervention effect and estimating the between-study heterogeneity variance in random-effects meta-analysis. In: Chandler J, McKenzie J, Boutron I, et al., eds.: Cochrane Methods. Cochrane Database of Systematic Reviews 2015.
37. Hartung J, Knapp G. A refined method for the meta-analysis of controlled clinical trials with binary outcome. *Stat Med* 2001;20(24):3875-89. [published Online First: 2002/01/10]
38. Sidik K, Jonkman JN. A simple confidence interval for meta-analysis. *Stat Med* 2002;21(21):3153-9. doi: 10.1002/sim.1262 [published Online First: 2002/10/11]
39. RDevelopmentCoreTeam. R: A language and environment for statistical computing. Vienna, Austria: R Foundation for Statistical Computing, 2013.
40. Viechtbauer W. Conducting Meta-Analyses in R with the metafor Package. 2010;36(3):48.
41. DerSimonian R, Laird N. Meta-analysis in clinical trials. *Control Clin Trials* 1986;7(3):177-88.

For peer review only

For peer review only - http://bmjopen.bmj.com/site/about/guidelines.xhtml

42. Salanti G, Higgins JP, Ades AE, et al. Evaluation of networks of randomized trials. *Stat Methods Med Res* 2008;17(3):279-301. doi: 10.1177/0962280207080643 [published Online First: 2007/10/09]
43. Higgins JP, Whitehead A. Borrowing strength from external trials in a meta-analysis. *Stat Med* 1996;15(24):2733-49. doi: 10.1002/(SICI)1097-0258(19961230)15:24<2733::AID-SIM562>3.0.CO;2-0
44. Jackson D, White IR, Riley RD. Quantifying the impact of between-study heterogeneity in multivariate meta-analyses. *Stat Med* 2012;31(29):3805-20. doi: 10.1002/sim.5453 [published Online First: 2012/07/06]
45. Efthimiou O, Mavridis D, Debray TP, et al. Combining randomized and non-randomized evidence in network meta-analysis. *Stat Med* 2017;36(8):1210-26. doi: 10.1002/sim.7223 [published Online First: 2017/01/14]
46. Salanti G, Ades AE, Ioannidis JP. Graphical methods and numerical summaries for presenting results from multiple-treatment meta-analysis: an overview and tutorial. *J Clin Epidemiol* 2011;64(2):163-71. doi: 10.1016/j.jclinepi.2010.03.016 [published Online First: 2010/08/05]
47. Veroniki AA, Straus SE, Fyraridis A, et al. The rank-heat plot is a novel way to present the results from a network meta-analysis including multiple outcomes. *J Clin Epidemiol* 2016;76:193-9. doi: 10.1016/j.jclinepi.2016.02.016 [published Online First: 2016/03/05]
48. Rucker G, Schwarzer G. Ranking treatments in frequentist network meta-analysis works without resampling methods. *BMC medical research methodology* 2015;15:58. doi: 10.1186/s12874-015-0060-8 [published Online First: 2015/08/01]
49. Rücker G, Schwarzer G, Krahn U, et al. netmeta: Network Meta-Analysis using Frequentist Methods. R package version 0.9-8. 2018 [Available from: https://CRAN.R-project.org/package=netmeta.
50. Plummer M. rjags: Bayesian Graphical Models using MCMC. R package version 4-8 2018 [Available from: https://CRAN.R-project.org/package=rjags.
51. Salanti G. Indirect and mixed-treatment comparison, network, or multiple-treatments meta-analysis: many names, many benefits, many concerns for the next generation evidence synthesis tool. *Res Synth Methods* 2012;3(2):80-97. doi: 10.1002/jrsm.1037 [published Online First: 2012/06/11]
52. Jansen JP, Naci H. Is network meta-analysis as valid as standard pairwise meta-analysis? It all depends on the distribution of effect modifiers. *BMC Med* 2013;11:159. doi: 10.1186/1741-7015-11-159 [published Online First: 2013/07/04]
53. Konig J, Krahn U, Binder H. Visualizing the flow of evidence in network meta-analysis and characterizing mixed treatment comparisons. *Stat Med* 2013;32(30):5414-29. doi: 10.1002/sim.6001 [published Online First: 2013/10/15]
54. White IR, Barrett JK, Jackson D, et al. Consistency and inconsistency in network meta-analysis: model estimation using multivariate meta-regression. *Res Synth Methods* 2012;3(2):111-25. doi: 10.1002/jrsm.1045
55. Veroniki AA, Mavridis D, Higgins JP, et al. Characteristics of a loop of evidence that affect detection and estimation of inconsistency: a simulation study. *BMC medical*

For peer review only

research methodology 2014;14:106. doi: 10.1186/1471-2288-14-106 [published Online First: 2014/09/23]
56. Song F, Clark A, Bachmann MO, et al. Simulation evaluation of statistical properties of methods for indirect and mixed treatment comparisons. *BMC medical research methodology* 2012;12:138. doi: 10.1186/1471-2288-12-138 [published Online First: 2012/09/14]
57. Turner RM, Davey J, Clarke MJ, et al. Predicting the extent of heterogeneity in meta-analysis, using empirical data from the Cochrane Database of Systematic Reviews. *Int J Epidemiol* 2012;41(3):818-27. doi: 10.1093/ije/dys041 [published Online First: 2012/03/31]
58. Chaimani A, Higgins JP, Mavridis D, et al. Graphical tools for network meta-analysis in STATA. *PLoS One* 2013;8(10):e76654. doi: 10.1371/journal.pone.0076654 [published Online First: 2013/10/08]
59. Chaimani A, Salanti G. Using network meta-analysis to evaluate the existence of small-study effects in a network of interventions. *Res Synth Methods* 2012;3(2):161-76. doi: 10.1002/jrsm.57 [published Online First: 2012/06/01]
60. Mavridis D, Efthimiou O, Leucht S, et al. Publication bias and small-study effects magnified effectiveness of antipsychotics but their relative ranking remained invariant. *J Clin Epidemiol* 2016;69:161-9. doi: 10.1016/j.jclinepi.2015.05.027 [published Online First: 2015/07/27]
61. Salanti G, Del Giovane C, Chaimani A, et al. Evaluating the quality of evidence from a network meta-analysis. *PLoS One* 2014;9(7):e99682. doi: 10.1371/journal.pone.0099682 [published Online First: 2014/07/06]

STATEMENTS

Declaration

The corresponding author had full access to all the data in the study and the final responsibility for the decision to submit for publication.
The senior author MK (the manuscript's guarantor) affirms that the manuscript is an honest, accurate, and transparent account of the study being reported and that no important aspects of the study have been omitted.

Author statement

The study was conceived and designed by MK, GS and EP. The protocol was drafted by AA, MK, AAV, OE, IK, GS and was revised critically for important intellectual content by all authors (AA, AAV, OE, IK, HN, SL, MP, PMH, PB, EP, GS, MK).

Acknowledgements

We thank the Jo's Trust for their assistance to involve public and patient representatives in this project.

Funding statement

For peer review only

This work is supported by National Institute for Health Research (NIHR) Research for Patient Benefits (P67307)(MK). It is also supported by the British Society of Colposcopy Cervical Pathology Jordan/Singer Award (P47773) and the Imperial College Healthcare Charity (P47907)(MK). AAV is funded by the European Union's Horizon 2020 (No 754936). None of the funders have any influence on the study design; in the collection, analysis, and interpretation of data; in the writing of the report; and in the decision to submit the article for publication.

Competing interests statement

We have read and understood BMJ policy on declaration of interests and declare that we have no conflict of interests. All authors have completed the ICMJE uniform disclosure form at www.icmje.org/coi_disclosure.pdf and declare: no support from any organisation for the submitted work; no financial relationships with any organisations that might have an interest in the submitted work in the previous three years; no other relationships or activities that could appear to have influenced the submitted work.
Dr Kyrgiou has received travel and conference expenses, honoraria and consultancy fees for commercial companies (Inovio, MSD etc); these activities are not related to the project.

Data sharing

No additional data available.

Copyright statement

"The Corresponding Author has the right to grant on behalf of all authors and does grant on behalf of all authors, a worldwide licence to the Publishers and its licensees in perpetuity, in all forms, formats and media (whether known now or created in the future), to i) publish, reproduce, distribute, display and store the Contribution, ii) translate the Contribution into other languages, create adaptations, reprints, include within collections and create summaries, extracts and/or, abstracts of the Contribution, iii) create any other derivative work(s) based on the Contribution, iv) to exploit all subsidiary rights in the Contribution, v) the inclusion of electronic links from the Contribution to third party material where-ever it may be located; and, vi) licence any third party to do any or all of the above."

For peer review only

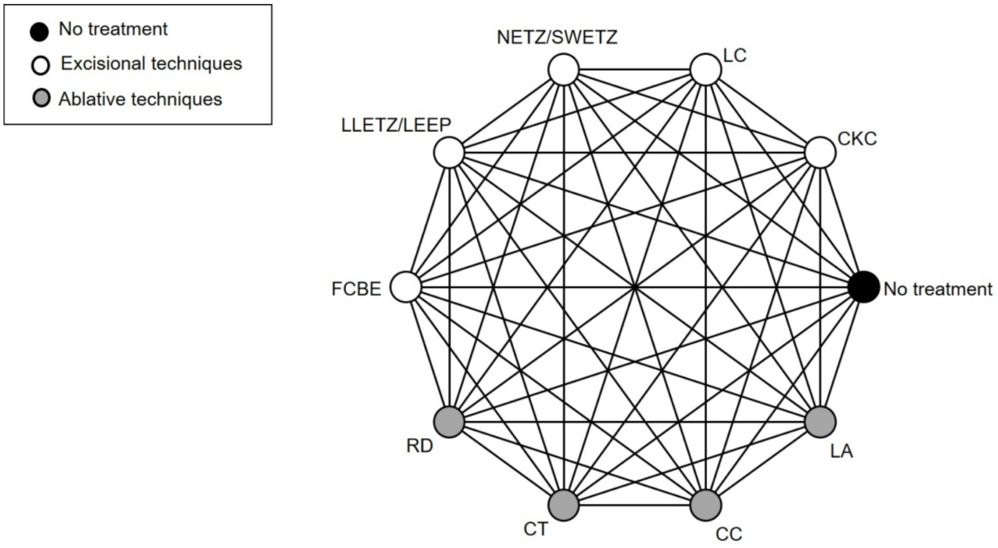

Network of possible pairwise comparisons between eligible treatment methods

203x112mm (300 x 300 DPI)

Supplementary File 1: Reporting checklist for protocol of a systematic review

Based on the PRISMA-P guidelines.

		Reporting Item	Page Number
Identification	#1a	Identify the report as a protocol of a systematic review	1
Update	#1b	If the protocol is for an update of a previous systematic review, identify as such	N/A (no previous NMA)
	#2	If registered, provide the name of the registry (such as PROSPERO) and registration number	2
Contact	#3a	Provide name, institutional affiliation, e-mail address of all protocol authors; provide physical mailing address of corresponding author	1
Contribution	#3b	Describe contributions of protocol authors and identify the guarantor of the review	15
	#4	If the protocol represents an amendment of a previously completed or published protocol, identify as such and list changes; otherwise, state plan for documenting important protocol amendments	4
Sources	#5a	Indicate sources of financial or other support for the review	15
Sponsor	#5b	Provide name for the review funder and / or sponsor	15
Role of sponsor or funder	#5c	Describe roles of funder(s), sponsor(s), and / or institution(s), if any, in developing the protocol	15
Rationale	#6	Describe the rationale for the review in the context of what is already known	3-4
Objectives	#7	Provide an explicit statement of the question(s) the review will address with reference to participants, interventions, comparators, and outcomes (PICO)	4
Eligibility criteria	#8	Specify the study characteristics (such as PICO, study design, setting, time frame) and report characteristics (such as years considered, language, publication status) to be used as criteria for eligibility for the review	4-5
Information sources	#9	Describe all intended information sources (such as electronic	5-6

For peer review only

		databases, contact with study authors, trial registers or other grey literature sources) with planned dates of coverage	
Search strategy	#10	Present draft of search strategy to be used for at least one electronic database, including planned limits, such that it could be repeated	5; Supplementary File 2
Study records - data management	#11a	Describe the mechanism(s) that will be used to manage records and data throughout the review	6
Study records - selection process	#11b	State the process that will be used for selecting studies (such as two independent reviewers) through each phase of the review (that is, screening, eligibility and inclusion in meta-analysis)	6
Study records - data collection process	#11c	Describe planned method of extracting data from reports (such as piloting forms, done independently, in duplicate), any processes for obtaining and confirming data from investigators	6
Data items	#12	List and define all variables for which data will be sought (such as PICO items, funding sources), any pre-planned data assumptions and simplifications	6
Outcomes and prioritization	#13	List and define all outcomes for which data will be sought, including prioritization of main and additional outcomes, with rationale	5
Risk of bias in individual studies	#14	Describe anticipated methods for assessing risk of bias of individual studies, including whether this will be done at the outcome or study level, or both; state how this information will be used in data synthesis	6-7
Data synthesis	#15a	Describe criteria under which study data will be quantitatively synthesised	7-8
	#15b	If data are appropriate for quantitative synthesis, describe planned summary measures, methods of handling data and methods of combining data from studies, including any planned exploration of consistency (such as I2, Kendall's τ)	7-9
	#15c	Describe any proposed additional analyses (such as sensitivity or subgroup analyses, meta-regression)	9
	#15d	If quantitative synthesis is not appropriate, describe the type of summary planned	N/A
Meta-bias(es)	#16	Specify any planned assessment of meta-bias(es) (such as	9

For peer review only

		publication bias across studies, selective reporting within studies)	
Confidence in cumulative evidence	#17	Describe how the strength of the body of evidence will be assessed (such as GRADE)	9

The PRISMA-P checklist is distributed under the terms of the Creative Commons Attribution License CC-BY 4.0. This checklist can be completed online using https://www.goodreports.org/, a tool made by the EQUATOR Network in collaboration with Penelope.ai

For peer review only

Supplementary File 2: Search algorithms

Medline Ovid

1 exp Uterine Cervical Neoplasms/
2 (cervi* and (cancer* or tumor* or tumour* or neoplas* or malignan* or carcinom*)).mp.
3 exp Cervical Intraepithelial Neoplasia/
4 CIN.mp.
5 (cervi* and (intraepithel* or epithel* or dysplasia or pre-cancer* or precancer*)).mp.
6 or/1-5
7 exp Conization/
8 (conisation or conization).mp.
9 exp Laser Therapy/
10 laser.mp.
11 exp Cryotherapy/
12 cryotherapy.mp.
13 cold coagulation.mp.
14 exp Diathermy/
15 diatherm*.mp.
16 cone biopsy.mp.
17 loop.mp.
18 LLETZ.mp.
19 LEEP.mp.
20 ablat*.mp.
21 excision*.mp.
22 transformation zone.mp.
23 (CKC or LA or LC or CC or RD or TZ).mp.
24 (conservative and (method* or treatment* or intervention* or management)).mp.
25 or/7-24
26 6 and 25
27 exp Premature Birth/
28 (preterm or premature).mp.
29 exp Infant, Low Birth Weight/
30 birth weight.mp.
31 Perinatal Mortality/
32 perinatal mortality.mp.
33 exp Intensive Care, Neonatal/
34 (neonatal and intensive care).mp.
35 exp Fertility/
36 fertil*.mp.
37 conception.mp.
38 exp Pregnancy/
39 pregnancy.mp.
40 gestation*.mp.
41 exp Abortion, Spontaneous/
42 miscarriage*.mp.
43 exp Cesarean Section/
44 (cesarean or caesarean).mp.
45 exp Obstetric Labor, Premature/
46 exp Labor, Obstetric/
47 (labor or labour).mp.

For peer review only

48 Fetal Membranes, Premature Rupture/
49 pPROM.mp.
50 or/27-49
51 26 and 50
key:
mp=title, original title, abstract, name of substance word, subject heading word

Embase Ovid

1 exp uterine cervix tumor/
2 (cervi* and (cancer* or tumor* or tumour* or neoplas* or malignan* or carcinom*)).mp.
3 uterine cervix carcinoma in situ/
4 CIN.mp.
5 (cervi* and (intraepithel* or epithel* or dysplasia or pre-cancer* or precancer*)).mp.
6 or/1-5
7 uterine cervix conization/
8 (conisation or conization).mp.
9 low level laser therapy/
10 laser.mp.
11 exp cryotherapy/
12 cryotherapy.mp.
13 cold coagulation.mp.
14 diathermy/
15 diatherm*.mp.
16 cone biopsy.mp.
17 loop.mp.
18 LLETZ.mp.
19 LEEP.mp.
20 ablat*.mp.
21 excision*.mp.
22 transformation zone.mp.
23 (CKC or LA or LC or CC or RD or TZ).mp.
24 (conservative and (method* or treatment* or intervention* or management)).mp.
25 or/7-24
26 6 and 25
27 prematurity/
28 (preterm or premature).mp.
29 exp low birth weight/
30 birth weight.mp.
31 perinatal mortality/
32 perinatal mortality.mp.
33 newborn intensive care/
34 (neonat* and intensive care).mp.
35 female fertility/
36 fertil*.mp.
37 conception/
38 conception.mp.
39 exp pregnancy/
40 pregnancy.mp.
41 gestation*.mp.
42 spontaneous abortion/

For peer review only

43 miscarriage*.mp.
44 cesarean section/
45 (cesarean or caesarean).mp.
46 premature labor/
47 (labor or labour).mp.
48 premature fetus membrane rupture/
49 pPROM.mp.
50 or/27-49
51 26 and 50
key:
mp=title, abstract, subject headings, heading word, drug trade name, original title, device manufacturer, drug manufacturer name

CENTRAL
#1 MeSH descriptor **Uterine Cervical Neoplasms** explode all trees
#2 cervi* and (cancer* or tumor* or tumour* or neoplas* or malignan* or carcinom*)
#3 MeSH descriptor **Cervical Intraepithelial Neoplasia** explode all trees
#4 CIN
#5 cervi* and (intraepithel* or epithel* or dysplasia or pre-cancer* or precancer*)
#6 (#1 OR #2 OR #3 OR #4 OR #5)
#7 MeSH descriptor **Conization** explode all trees
#8 conisation or conization
#9 MeSH descriptor **Laser Therapy** explode all trees
#10 laser
#11 MeSH descriptor **Cryotherapy** explode all trees
#12 cryotherapy
#13 cold coagulation
#14 MeSH descriptor **Diathermy** explode all trees
#15 diatherm*
#16 cone biopsy
#17 loop
#18 LLETZ
#19 LEEP
#20 ablat*
#21 excision*
#22 transformation zone
#23 CKC or LA or LC or CC or RD or TZ
#24 conservative and (method* or treatment* or intervention* or management)
#25 (#7 OR #8 OR #9 OR #10 OR #11 OR #12 OR #13 OR #14 OR #15 OR #16 OR #17 OR #18 OR #19 OR #20 OR #21 OR #22 OR #23 OR #24)
#26 (#6 AND #25)
#27 MeSH descriptor **Premature Birth** explode all trees
#28 preterm or premature
#29 MeSH descriptor **Infant, Low Birth Weight** explode all trees
#30 birth weight
#31 MeSH descriptor **Perinatal Mortality** explode all trees
#32 perinatal mortality
#33 MeSH descriptor **Intensive Care, Neonatal** explode all trees
#34 neonat* and (intensive care)
#35 MeSH descriptor **Fertility** explode all trees
#36 fertil*

For peer review only

#37 conception
#38 MeSH descriptor **Pregnancy** explode all trees
#39 pregnancy
#40 gestation*
#41 MeSH descriptor **Abortion, Spontaneous** explode all trees
#42 miscarriage*
#43 MeSH descriptor **Cesarean Section** explode all trees
#44 cesarean or caesarean
#45 MeSH descriptor **Obstetric Labor, Premature** explode all trees
#46 MeSH descriptor **Labor, Obstetric** explode all trees
#47 labor or labour
#48 MeSH descriptor **Fetal Membranes, Premature Rupture** explode all trees
#49 pPROM
#50 (#27 OR #28 OR #29 OR #30 OR #31 OR #32 OR #33 OR #34 OR #35 OR #36 OR #37 OR #38 OR #39 OR #40 OR #41 OR #42 OR #43 OR #44 OR #45 OR #46 OR #47 OR #48 OR #49)
#51 (#26 AND #50)

For peer review only

BMJ Open

Comparative fertility and pregnancy outcomes after local treatment for cervical intra-epithelial neoplasia and stage 1a1 cervical cancer: protocol for a systematic review and network meta-analysis from the CIRCLE Group

Journal:	*BMJ Open*
Manuscript ID	bmjopen-2018-028009.R1
Article Type:	Protocol
Date Submitted by the Author:	20-Jul-2019
Complete List of Authors:	Athanasiou, Antonios; Institute of Reproductive and Developmental Biology, Department of Surgery & Cancer, Faculty of Medicine, Imperial College London; Imperial College Healthcare NHS Trust<br>Veroniki, Areti Angeliki; Institute of Reproductive and Developmental Biology, Department of Surgery & Cancer, Faculty of Medicine, Imperial College London; Panepistimio Ioanninon, School of Education, Department of Primary Education<br>Efthimiou, Orestis; University of Bern, Institute of Social and Preventive Medicine (ISPM)<br>Kalliala, Ilkka; Institute of Reproductive and Developmental Biology, Department of Surgery & Cancer, Faculty of Medicine, Imperial College London; University of Helsinki and Helsinki University Hospital, Department of Obstetrics and Gynaecology<br>Naci, Huseyin; London School of Economics, Department of Health Policy<br>Bowden, Sarah; Institute of Reproductive and Developmental Biology, Department of Surgery & Cancer, Faculty of Medicine, Imperial College London; Imperial College Healthcare NHS Trust<br>Paraskevaidi, Maria; Institute of Reproductive and Developmental Biology, Department of Surgery & Cancer, Faculty of Medicine, Imperial College London<br>Martin-Hirsch, Pierre; Lancashire Teaching Hospitals NHS Foundation Trust, Department of Gynaecologic Oncology<br>Bennett, Philip; Institute of Reproductive and Developmental Biology, Department of Surgery & Cancer, Faculty of Medicine, Imperial College London; Imperial College Healthcare NHS Trust<br>Paraskevaidis, Evangelos; University of Ioannina and University Hospital of Ioannina, Department of Obstetrics & Gynaecology; Imperial College Healthcare NHS Trust<br>Salanti, Georgia; University of Bern, Institute of Social and Preventive Medicine (ISPM)<br>Kyrgiou, Maria; Institute of Reproductive and Developmental Biology, Department of Surgery & Cancer, Faculty of Medicine, Imperial College London; Imperial College Healthcare NHS Trust
**Primary Subject Heading**:	Obstetrics and gynaecology
Secondary Subject Heading:	Oncology, Evidence based practice

For peer review only - http://bmjopen.bmj.com/site/about/guidelines.xhtml

Keywords:	"Cervical Intraepithelial Neoplasia/surgery"[Mesh], "Cervical Intraepithelial Neoplasia/therapy"[Mesh], "Conization/adverse effects"[Mesh], "Ablation Techniques/adverse effects"[Mesh], "Pregnancy/complications"[Mesh], "Premature Birth"[Mesh

SCHOLARONE™
Manuscripts

Comparative fertility and pregnancy outcomes after local treatment for cervical intra-epithelial neoplasia and stage 1a1 cervical cancer: protocol for a systematic review and network meta-analysis from the CIRCLE Group

Antonios Athanasiou[1,2], Areti Angeliki Veroniki[1,3], Orestis Efthimiou[4], Ilkka Kalliala[1,5], Huseyin Naci[6], Sarah Bowden[1,2], Maria Paraskevaidi[1], Pierre Martin-Hirsch[7], Phillip Bennett[1,2], Evangelos Paraskevaidis[2,8], Georgia Salanti[4], Maria Kyrgiou[1,2]

[1]Institute of Reproductive and Developmental Biology, Department of Surgery & Cancer, Faculty of Medicine, Imperial College London, UK
[2]Imperial Healthcare NHS Trust, London, UK
[3]Department of Primary Education, School of Education, University of Ioannina, Greece
[4]Institute of Social and Preventive Medicine (ISPM), University of Bern, Mittelstrasse 43, 3012 Bern, Switzerland
[5]Department of Obstetrics and Gynaecology, University of Helsinki and Helsinki University Hospital, Finland
[6]Department of Health Policy, London School of Economics & Political Science, UK
[7]Department of Gynaecologic Oncology, Lancashire Teaching Hospitals, Preston, UK
[8]Department of Obstetrics & Gynaecology, University of Ioannina and University Hospital of Ioannina, Greece

AA: a.athanasiou@imperial.ac.uk; AAV: a.veroniki@imperial.ac.uk; OE: oremiou@gmail.com; IK: i.kalliala@imperial.ac.uk; HN: h.naci@lse.ac.uk; SB: s.lever@imperial.ac.uk; MP: m.paraskevaidi@imperial.ac.uk; PMH: pierre.martin-hirsch@lthtr.nhs.uk; PB: p.bennett@imperial.ac.uk; EP: eparaske@cc.uoi.gr; GS: georgia.salanti@ispm.unibe.ch; MK: m.kyrgiou@imperial.ac.uk

Corresponding author:
Maria Kyrgiou, MSc, PhD, MRCOG
3rd Floor, IRDB, Department of Surgery & Cancer
Imperial College London, Hammersmith Campus
Du Cane Road, W12 0NN, London
Email: m.kyrgiou@imperial.ac.uk - Tel: +44 2075942177

Word Count:

KEYWORDS
"Cervical Intraepithelial Neoplasia/surgery"[Mesh]
"Cervical Intraepithelial Neoplasia/therapy"[Mesh]
"Conization/adverse effects"[Mesh]
"Ablation Techniques/adverse effects"[Mesh]
"Pregnancy/complications"[Mesh]
"Premature Birth"[Mesh]

For peer review only

ABSTRACT

Introduction: There are several local treatment methods for cervical intra-epithelial neoplasia that remove or ablate a cone-shaped part of the uterine cervix. There is evidence to suggest that these increase the risk of preterm birth and that this is higher for techniques that remove larger parts of the cervix, although the data is conflicting. We present a protocol for a systematic review and network meta-analysis that will update the evidence and compare all treatments in terms of fertility and pregnancy complications.

Methods and Analysis: We will search electronic databases to identify randomised controlled trials (RCTs) and cohort studies comparing the fertility and pregnancy outcomes amongst different excisional and ablative treatment techniques and/or to untreated controls. The primary outcome will be preterm birth (PTB; <37weeks). Secondary outcomes will include severe or extreme PTB, prelabour rupture of membranes, low birth weight (<2500gr), neonatal intensive care unit admission, perinatal mortality, total pregnancy rates, 1st and 2nd trimester miscarriage. We will search for published and unpublished studies in electronic databases, trial registries and we will hand-search references of published papers. We will assess the risk of bias in RCTs and cohort studies using tools developed by the Cochrane Collaboration. Two investigators will independently assess the eligibility, abstract the data and assess the risk of bias of the identified studies. For each outcome, we will perform a meta-analysis for each treatment comparison and a network meta-analysis once the transitivity assumption holds, using the odds ratio for dichotomous data. We will use CINEMA to assess the quality of the evidence for the primary outcome.

Ethics and dissemination: Ethical approval is not required. Results will be disseminated to academic beneficiaries, medical practitioners, patients and the public.

PROSPERO registration number: CRD42018115495

ARTICLE SUMMARY

Strengths and limitations of this study

- This will be the first network meta-analysis to produce comprehensive summaries of the relative reproductive morbidity of treatment methods for cervical pre-invasive and early invasive disease.
- This network meta-analysis will compare all currently available treatment methods. Our results are expected to contribute to more effective patient counselling at colposcopy and antenatal clinics and may be utilised for the development of clinical guidelines.
- We will use state-of-the-art methods for combining randomised and non-randomised studies in a network meta-analysis.
- Risk of bias will be evaluated at both study and outcome level.
- One possible limitation of this review is that we expect to find mainly retrospective cohort studies at high risk of recall, selection and publication bias.

For peer review only - http://bmjopen.bmj.com/site/about/guidelines.xhtml

INTRODUCTION

The introduction of systematic call and recall screening programmes in the UK has resulted in a profound decrease in the incidence and mortality from cervical cancer, as pre-invasive precursors (cervical intra-epithelial neoplasia; CIN) can be detected by the screening programme and treated[1]. In England alone, 3.6 million women aged 25 to 64 attended for screening in 2013-14 and over 23,800 treatment procedures were carried out[2].

Local conservative treatment for cervical pre-invasive and early invasive disease removes or ablates a cone-shaped part of the cervix containing the precancerous cells. The choice of technique varies within the UK, across Europe and beyond. In some countries, knife excision (cold knife conisation; CKC) is still regularly performed; in others, ablation (LA) or conisation (LC) with the laser beam is common practice. In the UK, large loop excision of the transformation zone (LLETZ) is the preferred treatment, with some units offering alternative techniques more frequently than others. This preference is because LLETZ is quick, easy to do and of low cost.

The mean age of women undergoing CIN treatment is similar to the age of women having their first child. Although, previously, complications from treatment were thought to be relatively mild and uncommon, an increasing body of retrospective observational studies and meta-analyses suggested that treatment, particularly excision, adversely affects future reproduction and the risk of prematurity[3-8]. It has been suggested that the frequency and severity of the observed adverse events is higher for the more radical techniques and with increasing cone depth[4 7 9-14]. Preterm birth (PTB) is a major cause of neonatal death and disability and represents an enormous cost to the health services and the society in general.

Although all treatment techniques are highly effective in preventing recurrent pre-cancerous disease and future invasion[15], some of the data on the risk of reproductive morbidity for the treatment methods has been conflicting[3 5 7 10 16-18] and their comparative reproductive morbidity remains unclear. With some authors raising concerns that the progressive reduction in the radicality of treatment has led to increased risk of future post-treatment invasive disease[19 20], and others advocating the move to less radical techniques, such as laser ablation, for the prevention of treatment-associated adverse obstetric outcomes, such as preterm birth or perinatal mortality[4 21], high-quality synthesis of the current evidence base is an urgent unmet need. Given the premalignant nature of the condition, an RCT comparing the various treatment technique to no treatment will never be conducted.

The quantification of the comparative reproductive morbidity of different treatment techniques and cone lengths has become a women's health priority. This requires high-quality synthesis of the evidence in comprehensive summaries that will become available for effective patient counselling at colposcopy and antenatal clinics for patients, clinicians and policy makers. A clinical ranking of treatments with regards to the risk of preterm birth may allow the quantification of risk and the detection of women at high risk of PTB that would benefit from intensive surveillance antenatally, while minimising the unnecessary interventions for those at lower risk.

For peer review only - http://bmjopen.bmj.com/site/about/guidelines.xhtml

The key methodological vehicle to synthesize evidence is systematic reviews and their quantitative component, meta-analysis. Network meta-analysis (NMA) is an extension of pairwise meta-analysis, which can be used to estimate the relative effectiveness of several competing treatments. An NMA has never been used before to assess the comparative efficacy and harms of the different treatment techniques in this field. For every treatment comparison NMA synthesizes both direct evidence (i.e. coming from studies comparing head-to-head the treatments of interest) and indirect evidence (i.e. coming from studies comparing the treatments of interest via an intermediate common comparator)[22-25]. In addition, NMA allows the estimation of relative effects between all available treatments, can lead to an increased precision as compared to the pairwise meta-analysis and provides a ranking of the available competing treatments. The potential of NMA has been recognized by the National Institute of Clinical Excellence (NICE)[26] and several international Health Technology Assessment (HTA) agencies[27 28].

The objective of this systematic review and NMA is to update the evidence and to compare the various local treatment methods to manage CIN in terms of fertility, early (<24 weeks of gestation) and late (>24 weeks) pregnancy complications. This is part of the CIRCLE project (**C**ervical Cancer Incidence, C**I**N **R**ecurrence and **R**eproduction after **L**ocal **E**xcision), which aims to generate a clinically useful raking of alternative options for treatment of CIN according to their efficacy (risk of pre-invasive and invasive recurrence), morbidity and cost-effectiveness.

METHODS AND ANALYSIS

This protocol complies with the Preferred Reporting Items for Systematic Review and Meta-Analysis Protocols (PRISMA-P) (Supplementary File 1)[29]. Any changes in this protocol will be recorded in an updated version of the PROSPERO registration.

Criteria for considering studies for this review

Types of participants

We will include women of all ages with a prior history of local surgical treatment for CIN or micro-invasive early cervical cancer (stage IA1). Their status can be confirmed with histological or cytological diagnosis and irrespective of the grade of the treated lesion for both squamous and glandular intra-epithelial neoplasia. Studies recruiting solely women at high risk of preterm birth (such as previous history of preterm birth) or studies including only patients treated during pregnancy will be excluded.

Types of interventions

We aim to compare 9 different excisional or ablative techniques used for conservative treatment for cervical intraepithelial neoplasia (CIN). The excisional techniques include cold knife conisation (CKC); laser conisation (LC); needle excision of the transformation zone (NETZ), also known as straight wire excision of the transformation zone (SWETZ); large loop excision of the transformation zone (LLETZ), also known as loop electrosurgical

excisional procedure (LEEP); Fischer cone biopsy excision (FCBE). The ablative techniques include radical point diathermy (RD); cryotherapy (CT); cold coagulation (CC); laser ablation (LA). We will include studies comparing any of these treatments with each other or with no treatment. Figure 1 shows a network example of all possible comparisons between eligible interventions for the primary analysis. If the specific treatment technique is not specified, these will be grouped under the broader categories excision (ENS) or ablation (ANS).

Outcome measures

Primary outcome

- PTB, defined as less than 37 weeks of gestation

Secondary outcomes

- Spontaneous PTB (sPTB), defined as less than 37 weeks of gestation
- Severe PTB, defined as less than 32/34 weeks of gestation
- Extreme PTB, defined as less than 28/30 weeks of gestation
- PROM (Prelabour Rupture of Membranes) defined as membrane rupture before the onset of labour
- Low Birth Weight (LBW), defined as infant born weighing less than 2500g (late neonatal obstetric outcome)
- Neonatal Intensive Care Unit (NICU) admission
- Perinatal mortality, defined as the number of stillbirths and neonatal deaths occurring within 28 days after birth
- Total pregnancy rate, defined as any pregnancy occurring from CIN treatment till study completion irrespective of outcome (miscarriage, ectopic, molar pregnancy, termination of pregnancy, live birth, stillbirth)'.
- Rates of women requiring more than 12 months to conceive
- 1st trimester miscarriage, defined as miscarriage at less than 12 weeks of gestation
- 2nd trimester miscarriage, defined as miscarriage between 12-24 weeks of gestation

Outcomes and their classification into primary or secondary were decided after clinical experts' opinion. Total pregnancy rate will be recorded for the whole study period and/or prespecified intervals, if data is available.

Types of studies

We will include randomised controlled trials (RCTs), quasi-RCTs and cohort studies comparing fertility and early (<24 weeks of gestation) or late (>24 weeks of gestation) pregnancy outcomes amongst surgical techniques and those that compare to no treatment. Studies that do not perform a comparison between treatments (i.e. "single-arm studies") will be excluded. Studies that compared a treatment with an untreated group will be included irrespective of the type of the untreated group (e.g. studies that used data from untreated women from the general population; studies that used self-matching, i.e. including women with pregnancies before and after treatment; studies that used data from women with a

For peer review only - http://bmjopen.bmj.com/site/about/guidelines.xhtml

history of abnormal cytology/HPV infection/untreated CIN). There will be no time or language restriction.

Information sources and search strategy

An experienced librarian will search The Cochrane Gynaecological Cancer Specialised Register; Cochrane Central Register of Controlled Trials (CENTRAL); MEDLINE; and EMBASE for eligible studies from inception. The search algorithms for these databases are presented in Supplementary File 2. We will search Metaregister, Physicians Data Query, www.controlled-trials.com/rct, www.clinicaltrials.gov and www.cancer.gov/clinicaltrials for ongoing studies. There will be no time or language restriction.

We will contact the corresponding author of any relevant ongoing trials for further information and unpublished data. In an attempt to identify any articles missed by the initial search, we will use the 'related articles' feature in MEDLINE. We will also hand search the references of the retrieved articles and meta-analyses. We will search conference proceedings and abstracts through ZETOC (http://zetoc.mimas.ac.uk), and theses through WorldCat Dissertations. The selected conferences will include: British Society of Colposcopy and Cervical Pathology; International Federation of Cervical Pathology and Colposcopy; Annual Meeting of European Federation of Colposcopy; Annual Meeting of the American Society of Colposcopy and Cervical Pathology. We will contact experts in the field, including directors of UK cancer and colposcopy registries, to identify further reports of studies. We will include both published and unpublished studies.

Study selection

We will download abstracts retrieved into a reference management software, Zotero. Then, two persons will independently review titles and abstracts retrieved by the search (level 1). At level 2, we will obtain the full text of all included articles and two reviewers will independently use the same inclusion criteria to determine eligibility. Disagreements at any level will be resolved via discussion with a third member of the review team.

Data collection

Two reviewers will extract data independently using a standardised data collection form in Excel. Disagreements will be resolved through discussion. Information extracted will include study characteristics (such as author, publication year and study design), participants (such as age, CIN grade and smoking) and comparison group characteristics, setting, inclusion/exclusion criteria, intervention details, outcome measures and dropout rates. In RCTs we will prefer arm-level data (number of events and sample size per intervention arm), but if these are missing, the study-level data will be used in the analysis, e.g. reported odds ratios (ORs) and a measure of their uncertainty (e.g. confidence interval). In observational studies we will extract estimates of treatment effects that are adjusted for the lack of randomisation, i.e. after taking into account the impact of potential confounders, or, if these are missing, the reported unadjusted estimates, as well as the corresponding uncertainty measure.

For peer review only - http://bmjopen.bmj.com/site/about/guidelines.xhtml

Risk of bias assessment

Results of the meta-analyses will be interpreted in light of risk of bias assessment of the included studies. Two investigators will independently assess the methodological quality/risk of bias of the studies that fulfil the eligibility criteria and differences will be resolved by discussion with a third investigator.

For RCTs, the risk of bias will be assessed using the tool[30] developed by Cochrane assessing the following domains: randomisation process, deviations from the intended interventions, missing outcome data, measurement of the outcome and selection of the reported result. The risk of bias in each domain, as well as the overall risk of bias, will be rated as "low risk", "some concerns", or "high risk", after answering the signalling questions of each domain with "Yes", "Potentially Yes", "Potentially No" or "No". When inadequate details are reported in the study to be able to rate a risk of bias item, we will contact the study authors for additional information.

For non-randomised studies (NRS), we will use the ROBINS-I tool[31] developed by the Cochrane collaboration that facilitates the evaluation of the risk of bias by considering that each NRS is an attempt to mimic an RCT comparing the effects of the intervention or exposure studied. During the review stage, we will evaluate the risk of bias in the following domains: confounding, selection of participants into the study, classification of interventions, deviations from intended interventions, missing data, measurement of outcomes and selection of the reported results. The confounding factors that we will evaluate are age, parity and smoking. Each ROBINS-I domain and the overall risk of bias will be assessed as "low", "moderate", "serious", or "critical", after answering the signalling questions of each domain with "Yes", "Potentially Yes", "Potentially No" or "No".

Statistical synthesis

Characteristics of included studies and Network

We will generate descriptive statistics for eligible studies and study population characteristics, describing the types of comparisons and important clinical or methodological variables (such as publication year, study design and source of data). We will present the evidence in a network diagram per outcome. The total number of patients will be reflected in the size of the nodes, while the weight of each edge will be proportional to the number of studies per treatment comparison.

Pairwise meta-analyses

We will synthesise data to obtain summary ORs for dichotomous outcomes with a 95% CI in an inverse variance random-effects model assuming that the studies are estimating different but related treatment effects. In each meta-analysis, we will estimate the between-study variance with the restricted maximum likelihood estimator[32 33] and its 95% CI using the Q-profile approach[34]. We will assess between-study variance using the I^2 statistic along a 95% confidence interval (CI)[35 36]. We will estimate each summary effect size and its 95% CI using the Hartung-Knapp-Sidik-Jonkman method[32 37 38] to handle meta-analyses that include a

For peer review only - http://bmjopen.bmj.com/site/about/guidelines.xhtml

small number of studies. All meta-analyses will be conducted in R[39] using the *metafor* package[40].

Network meta-analyses (NMA)

Data synthesis

We will fit a random effects NMA model, taking into account the correlation induced by multi-arm studies[41]. We will use a random-effects model, since we anticipate methodological and clinical heterogeneity across studies. We will assume a common between-study variance parameter for all comparisons in the network, so that treatment comparisons informed by a single study can borrow strength from the remaining network[42 43]. Clinically, this assumption is reasonable because all treatments included in the network are of the same nature. We will estimate the common between-study variance with the DerSimonian and Laird method of moments approach[44].

We expect that we will include several NRS in our dataset. In that case, we will employ the methods described by Efthimiou et al.[45], i.e. a “design-adjusted”, and a three-level hierarchical NMA model. Using these methods, we will incorporate the totality of available information, both randomized and observational, in a joint NMA. We will start by analysing the study-specific estimates from the NRS at face value. Then, in extensive sensitivity analyses we will explore the impact of assigning different levels of credibility and subsequently down-weighting the NRS, according to experts’ opinion and the risk of bias as assessed in ROBINS-I.

For each treatment comparison we will report the estimated OR, the 95% CI and the 95% prediction intervals. We will also estimate the ranking probabilities for all treatments of being at each possible rank for each intervention. Thus, we will obtain a treatment hierarchy using the surface under the cumulative ranking curve (SUCRA) or P-scores and mean ranks[46]. A rank-heat plot will be used to depict the SUCRA values or P-scores for all outcomes[47 48]. All NMAs will be fit in R[39] with the *netmeta*[49] and *rjags*[50] package.

We will repeat our NMAs after grouping all excisional and all ablative techniques together; see groupings of treatments in Figure 1.

Assessment of the transitivity assumption

NMA rests on the assumption of transitivity, i.e. that effect modifiers have a similar distribution across treatment comparisons in a network[24 51 52]. In order to assess the plausibility of this assumption, we will summarize study and patient-level characteristics that are expected to influence relative treatment effects, for each pairwise comparison for which direct evidence is available in the network. In this NMA, the most important effect modifiers are expected to be year of study, method of ascertainment of exposure/outcome (hospital records, registries or interviews/questionnaires), age, parity, smoking and CIN grade. We will visually inspect the similarity of the identified studies in terms of these effect modifiers. We will investigate the inclusion and exclusion criteria of all studies, to make sure that patients,

For peer review only

treatments and outcomes in the studies are sufficiently similar in all aspects that are expected to modify relative treatment effects.

Assessment of statistical inconsistency

Checking the network for inconsistency offers an additional way of assessing the validity of the transitivity assumption. In order to evaluate the presence of inconsistency locally, we will separate the indirect from the direct evidence for each comparison and infer about their differences following the back-calculation method[53]. We will also follow a global approach for assessing consistency in the network, by applying the design-by-treatment interaction model[54]. Simulations suggest that inconsistency tests have low power to detect true inconsistency[55 56]. Therefore, we will conceptually assess the transitivity assumption (see previous paragraph) even in the absence of evidence for inconsistency. We will perform both local (back-calculation method) and global (design-by-treatment interaction model) assessments in R[39] using the *netmeta* package[49].

When a network includes both randomised and non-randomised evidence, we will also explore differences between the different types of evidence, as discussed in Efthimiou et al[45]. For each treatment comparison there may be up to four different types of evidence: direct randomised, indirect randomised, direct non-randomised and indirect non-randomised. We will summarise all evidence by type, for each treatment comparison. If data permits, important discrepancies between these types will be further investigated, as they might indicate a breach of the transitivity assumption (e.g. when randomised and non-randomised evidence are very different in terms of populations, interventions, etc.), or the presence of important, unaccounted confounding in the non-randomised evidence. If a source of disagreement is identified, it will be included in our analysis through network meta-regression models[23].

Exploring heterogeneity and inconsistency: subgroup analyses, meta-regression, and sensitivity analyses

We will assess the extent of statistical heterogeneity by inspecting the 95% prediction intervals and by comparing the estimated value of the between-study variance with the empirical distribution derived by Turner et al for dichotomous data[57]. For the primary outcome, we will explore the following possible sources of heterogeneity and inconsistency: year of study, method of ascertainment of exposure/outcome (hospital records, registries or interviews/questionnaires), age, parity, smoking and CIN grade. If sufficient studies are available, the role of these variables will be explored by means of subgroup analyses (categorical characteristics) or network meta-regressions (continuous characteristics).

Reporting bias and small-study effects

In order to assess possible existence of small studies giving different effect estimates than larger studies, we will visually explore the funnel-plots for each treatment against the untreated group (using the relevant studies) when at least 10 studies inform the underling treatment comparison. We will also assess for small-study effects using the comparison

For peer review only - http://bmjopen.bmj.com/site/about/guidelines.xhtml

adjusted funnel plot[58] and will conduct a network meta-regression using the study variance as a covariate[59 60].

Assessment of the credibility of the evidence

We will evaluate the credibility of the evidence contributing to the network estimates in the primary outcome using CINEMA[61] (http://cinema.ispm.ch/). Two team members will determine the degree of confidence in the estimated NMA results by assessing the six CINEMA domains: within-study bias (i.e., risk of bias in the included studies), across-study bias (i.e., publication and reporting bias), indirectness, imprecision, heterogeneity and incoherence (i.e., differences between direct and indirect evidence)[61]. Each network summary estimate will initially be judged as high quality, but it will be downgraded if this is judged appropriate according to the six domains. Judgements within each domain will be summarized for each NMA relative treatment effect as: very low, low, moderate or high.

Patient and Public involvement

We have discussed the project with Jo's Cervical Cancer Trust (a UK charity who supports patients affected by cervical pre-invasive or invasive cervical disease and campaigns for excellence in cervical cancer treatment and prevention).
Two patient representatives through Jo's Trust with personal experience of cervical disease have assisted us in the design of the study and the development of research questions. We aim to recruit more patients through Jo's Voice, who will help us understand the key priorities from patients' perspective, produce lay summaries and disseminate the results to the wider public.

ETHICS AND DISSEMINATION

This review does not require ethical approval. We identified four groups of potential stakeholders (academic beneficiaries; health-related agencies and decision-makers; medical practitioners; patients and public) and specific action items to effectively target them. We will publish papers in influential open access journals and we will present data at high-profile conferences. We will make the datasets available to the wider research community. We will organise a workshop with key stakeholders. We will develop information sheets and briefings, highlighting the key findings and circulate newsletters. We will engage the press with presentations and social media interviews and we will work closely with Jo's Trust charity that plays an important role in educating patient communities.

Figure 1. Network of possible pairwise comparisons between eligible treatment methods
Abbreviations: **FCBE**=Fischer cone biopsy excision; **LLETZ**= large loop excision of the transformation zone, also known as **LEEP**=loop electrosurgical excisional procedure; **NETZ**=needle excision of the transformation, also known as **SWETZ**=straight wire excision of the transformation zone; **LC**=laser conisation; **CKC**=cold knife conisation; **LA**=laser ablation; **CC**=cold coagulation; **CT**=cryotherapy; **RD**=radical point diathermy

For peer review only - http://bmjopen.bmj.com/site/about/guidelines.xhtml

REFERENCES

1. Quinn M, Babb P, Jones J, et al. Effect of screening on incidence of and mortality from cancer of cervix in England: evaluation based on routinely collected statistics. *BMJ* 1999;318(7188):904-8.

2. PublicHealthEngland. Cervical screening: programme overview. http://www.cancerscreening.nhs.uk/cervical, 2015.

3. Kyrgiou M, Koliopoulos G, Martin-Hirsch P, et al. Obstetric outcomes after conservative treatment for intraepithelial or early invasive cervical lesions: systematic review and meta-analysis. *Lancet* 2006;367(9509):489-98. doi: 10.1016/s0140-6736(06)68181-6 [published Online First: 2006/02/14]

4. Arbyn M, Kyrgiou M, Simoens C, et al. Perinatal mortality and other severe adverse pregnancy outcomes associated with treatment of cervical intraepithelial neoplasia: meta-analysis. *BMJ* 2008;337:a1284. doi: 10.1136/bmj.a1284 [published Online First: 2008/09/20]

5. Kyrgiou M, Mitra A, Arbyn M, et al. Fertility and early pregnancy outcomes after treatment for cervical intraepithelial neoplasia: systematic review and meta-analysis. *Bmj* 2014;349:g6192. doi: 10.1136/bmj.g6192 [published Online First: 2014/10/30]

6. Kyrgiou M, Mitra A, Arbyn M, et al. Fertility and early pregnancy outcomes after conservative treatment for cervical intraepithelial neoplasia. *Cochrane Database Syst Rev* 2015(9):Cd008478. doi: 10.1002/14651858.CD008478.pub2 [published Online First: 2015/09/30]

7. Kyrgiou M, Athanasiou A, Paraskevaidi M, et al. Adverse obstetric outcomes after local treatment for cervical preinvasive and early invasive disease according to cone depth: systematic review and meta-analysis. *Bmj* 2016;354:i3633. doi: 10.1136/bmj.i3633 [published Online First: 2016/07/30]

8. Kyrgiou M, Athanasiou A, Kalliala IEJ, et al. Obstetric outcomes after conservative treatment for cervical intraepithelial lesions and early invasive disease. *Cochrane Database Syst Rev* 2017;11:Cd012847. doi: 10.1002/14651858.Cd012847 [published Online First: 2017/11/03]

9. Kyrgiou M, Arbyn M, Martin-Hirsch P, et al. Increased risk of preterm birth after treatment for CIN. *Bmj* 2012;345:e5847. doi: 10.1136/bmj.e5847 [published Online First: 2012/09/07]

10. Castanon A, Landy R, Brocklehurst P, et al. Risk of preterm delivery with increasing depth of excision for cervical intraepithelial neoplasia in England: nested case-control study. *BMJ* 2014;349:g6223. [published Online First: 2014/11/05]

11. Noehr B, Jensen A, Frederiksen K, et al. Depth of cervical cone removed by loop electrosurgical excision procedure and subsequent risk of spontaneous preterm delivery. *Obstet Gynecol* 2009;114(6):1232-8. doi: 10.1097/AOG.0b013e3181bf1ef2 [published Online First: 2009/11/26]

12. Khalid S, Dimitriou E, Conroy R, et al. The thickness and volume of LLETZ specimens can predict the relative risk of pregnancy-related morbidity. *Bjog* 2012;119(6):685-91. doi: 10.1111/j.1471-0528.2011.03252.x [published Online First: 2012/02/15]

For peer review only - http://bmjopen.bmj.com/site/about/guidelines.xhtml

13. Founta C, Arbyn M, Valasoulis G, et al. Proportion of excision and cervical healing after large loop excision of the transformation zone for cervical intraepithelial neoplasia. *Bjog* 2010;117(12):1468-74. doi: 10.1111/j.1471-0528.2010.02709.x [published Online First: 2010/09/16]
14. Kyrgiou M, Valasoulis G, Stasinou SM, et al. Proportion of cervical excision for cervical intraepithelial neoplasia as a predictor of pregnancy outcomes. *Int J Gynaecol Obstet* 2015;128(2):141-7. doi: 10.1016/j.ijgo.2014.07.038 [published Online First: 2014/12/03]
15. Martin-Hirsch PP, Paraskevaidis E, Bryant A, et al. Surgery for cervical intraepithelial neoplasia. *Cochrane Database Syst Rev* 2013(12):Cd001318. doi: 10.1002/14651858.CD001318.pub3 [published Online First: 2013/12/05]
16. Conner SN, Frey HA, Cahill AG, et al. Loop electrosurgical excision procedure and risk of preterm birth: a systematic review and meta-analysis. *Obstet Gynecol* 2014;123(4):752-61. doi: 10.1097/AOG.0000000000000174 [published Online First: 2014/05/03]
17. Danhof NA, Kamphuis EI, Limpens J, et al. The risk of preterm birth of treated versus untreated cervical intraepithelial neoplasia (CIN): a systematic review and meta-analysis. *Eur J Obstet Gynecol Reprod Biol* 2015;188:24-33. doi: 10.1016/j.ejogrb.2015.02.033 [published Online First: 2015/03/17]
18. Jin G, LanLan Z, Li C, et al. Pregnancy outcome following loop electrosurgical excision procedure (LEEP) a systematic review and meta-analysis. *Arch Gynecol Obstet* 2014;289(1):85-99. doi: 10.1007/s00404-013-2955-0 [published Online First: 2013/07/12]
19. Strander B, Hällgren J, Sparén P. Effect of ageing on cervical or vaginal cancer in Swedish women previously treated for cervical intraepithelial neoplasia grade 3: population based cohort study of long term incidence and mortality. *BMJ* 2014;348:f7361. [published Online First: 2014/01/14]
20. Arbyn M, Kyrgiou M, Gondry J, et al. Long term outcomes for women treated for cervical precancer. *Bmj* 2014;348:f7700. doi: 10.1136/bmj.f7700 [published Online First: 2014/01/16]
21. Paraskevaidis E, Kyrgiou M, Martin-Hirsch P. Have we dismissed ablative treatment too soon in colposcopy practice? *Bjog* 2007;114(1):3-4. doi: 10.1111/j.1471-0528.2006.01178.x [published Online First: 2007/01/20]
22. Caldwell DM, Ades AE, Higgins JP. Simultaneous comparison of multiple treatments: combining direct and indirect evidence. *BMJ* 2005;331(7521):897-900. doi: 10.1136/bmj.331.7521.897
23. Salanti G, Marinho V, Higgins JP. A case study of multiple-treatments meta-analysis demonstrates that covariates should be considered. *J Clin Epidemiol* 2009;62(8):857-64. doi: 10.1016/j.jclinepi.2008.10.001 [published Online First: 2009/01/20]
24. Cipriani A, Higgins JP, Geddes JR, et al. Conceptual and technical challenges in network meta-analysis. *Ann Intern Med* 2013;159(2):130-7. doi: 10.7326/0003-4819-159-2-201307160-00008

For peer review only

25. Efthimiou O, Debray TP, van Valkenhoef G, et al. GetReal in network meta-analysis: a review of the methodology. *Res Synth Methods* 2016;7(3):236-63. doi: 10.1002/jrsm.1195 [published Online First: 2016/01/13]
26. NICE. Developing NICE guidelines: the manual. Process and methods (PMG20). Reviewing research evidence 2017 [Available from: https://www.nice.org.uk/process/pmg20/chapter/reviewing-research-evidence.
27. Caldwell DM, Dias S, Welton NJ. Extending Treatment Networks in Health Technology Assessment: How Far Should We Go? *Value Health* 2015;18(5):673-81. doi: 10.1016/j.jval.2015.03.1792 [published Online First: 2015/08/25]
28. CADTH. Network Meta-Analysis 2015 [Available from: https://www.cadth.ca/network-meta-analysis.
29. Moher D, Shamseer L, Clarke M, et al. Preferred reporting items for systematic review and meta-analysis protocols (PRISMA-P) 2015 statement. *Syst Rev* 2015;4:1. doi: 10.1186/2046-4053-4-1 [published Online First: 2015/01/03]
30. Higgins JPT, Sterne JAC, Savović J, et al. A revised tool for assessing risk of bias in randomized trials. In: Chandler J, McKenzie J, Boutron I, et al., eds.: Cochrane Methods. Cochrane Database of Systematic Reviews 2016.
31. Sterne JA, Hernan MA, Reeves BC, et al. ROBINS-I: a tool for assessing risk of bias in non-randomised studies of interventions. *BMJ* 2016;355:i4919. doi: 10.1136/bmj.i4919 [published Online First: 2016/10/14]
32. Veroniki AA, Jackson D, Viechtbauer W, et al. Methods to estimate the between-study variance and its uncertainty in meta-analysis. *Res Synth Methods* 2016;7(1):55-79. doi: 10.1002/jrsm.1164 [published Online First: 2015/09/04]
33. Langan D, Higgins JPT, Jackson D, et al. A comparison of heterogeneity variance estimators in simulated random-effects meta-analyses. *Res Synth Methods* 2018 doi: 10.1002/jrsm.1316 [published Online First: 2018/08/02]
34. Viechtbauer W. Confidence intervals for the amount of heterogeneity in meta-analysis. *Stat Med* 2007;26(1):37-52. doi: 10.1002/sim.2514 [published Online First: 2006/02/08]
35. Higgins JP, Thompson SG, Deeks JJ, et al. Measuring inconsistency in meta-analyses. *BMJ* 2003;327(7414):557-60. doi: 10.1136/bmj.327.7414.557 [published Online First: 2003/09/06]
36. Veroniki AA, Jackson D, Viechtbauer W, et al. Recommendations for quantifying the uncertainty in the summary intervention effect and estimating the between-study heterogeneity variance in random-effects meta-analysis. In: Chandler J, McKenzie J, Boutron I, et al., eds.: Cochrane Methods. Cochrane Database of Systematic Reviews 2015.
37. Hartung J, Knapp G. A refined method for the meta-analysis of controlled clinical trials with binary outcome. *Stat Med* 2001;20(24):3875-89. [published Online First: 2002/01/10]
38. Sidik K, Jonkman JN. A simple confidence interval for meta-analysis. *Stat Med* 2002;21(21):3153-9. doi: 10.1002/sim.1262 [published Online First: 2002/10/11]
39. RDevelopmentCoreTeam. R: A language and environment for statistical computing. Vienna, Austria: R Foundation for Statistical Computing, 2013.

For peer review only - http://bmjopen.bmj.com/site/about/guidelines.xhtml

40. Viechtbauer W. Conducting Meta-Analyses in R with the metafor Package. 2010;36(3):48.
41. DerSimonian R, Laird N. Meta-analysis in clinical trials. *Control Clin Trials* 1986;7(3):177-88.
42. Salanti G, Higgins JP, Ades AE, et al. Evaluation of networks of randomized trials. *Stat Methods Med Res* 2008;17(3):279-301. doi: 10.1177/0962280207080643 [published Online First: 2007/10/09]
43. Higgins JP, Whitehead A. Borrowing strength from external trials in a meta-analysis. *Stat Med* 1996;15(24):2733-49. doi: 10.1002/(SICI)1097-0258(19961230)15:24<2733::AID-SIM562>3.0.CO;2-0
44. Jackson D, White IR, Riley RD. Quantifying the impact of between-study heterogeneity in multivariate meta-analyses. *Stat Med* 2012;31(29):3805-20. doi: 10.1002/sim.5453 [published Online First: 2012/07/06]
45. Efthimiou O, Mavridis D, Debray TP, et al. Combining randomized and non-randomized evidence in network meta-analysis. *Stat Med* 2017;36(8):1210-26. doi: 10.1002/sim.7223 [published Online First: 2017/01/14]
46. Salanti G, Ades AE, Ioannidis JP. Graphical methods and numerical summaries for presenting results from multiple-treatment meta-analysis: an overview and tutorial. *J Clin Epidemiol* 2011;64(2):163-71. doi: 10.1016/j.jclinepi.2010.03.016 [published Online First: 2010/08/05]
47. Veroniki AA, Straus SE, Fyraridis A, et al. The rank-heat plot is a novel way to present the results from a network meta-analysis including multiple outcomes. *J Clin Epidemiol* 2016;76:193-9. doi: 10.1016/j.jclinepi.2016.02.016 [published Online First: 2016/03/05]
48. Rucker G, Schwarzer G. Ranking treatments in frequentist network meta-analysis works without resampling methods. *BMC medical research methodology* 2015;15:58. doi: 10.1186/s12874-015-0060-8 [published Online First: 2015/08/01]
49. Rücker G, Schwarzer G, Krahn U, et al. netmeta: Network Meta-Analysis using Frequentist Methods. R package version 0.9-8. 2018 [Available from: https://CRAN.R-project.org/package=netmeta.
50. Plummer M. rjags: Bayesian Graphical Models using MCMC. R package version 4-8 2018 [Available from: https://CRAN.R-project.org/package=rjags.
51. Salanti G. Indirect and mixed-treatment comparison, network, or multiple-treatments meta-analysis: many names, many benefits, many concerns for the next generation evidence synthesis tool. *Res Synth Methods* 2012;3(2):80-97. doi: 10.1002/jrsm.1037 [published Online First: 2012/06/11]
52. Jansen JP, Naci H. Is network meta-analysis as valid as standard pairwise meta-analysis? It all depends on the distribution of effect modifiers. *BMC Med* 2013;11:159. doi: 10.1186/1741-7015-11-159 [published Online First: 2013/07/04]
53. Konig J, Krahn U, Binder H. Visualizing the flow of evidence in network meta-analysis and characterizing mixed treatment comparisons. *Stat Med* 2013;32(30):5414-29. doi: 10.1002/sim.6001 [published Online First: 2013/10/15]

For peer review only - http://bmjopen.bmj.com/site/about/guidelines.xhtml

54. White IR, Barrett JK, Jackson D, et al. Consistency and inconsistency in network meta-analysis: model estimation using multivariate meta-regression. *Res Synth Methods* 2012;3(2):111-25. doi: 10.1002/jrsm.1045
55. Veroniki AA, Mavridis D, Higgins JP, et al. Characteristics of a loop of evidence that affect detection and estimation of inconsistency: a simulation study. *BMC medical research methodology* 2014;14:106. doi: 10.1186/1471-2288-14-106 [published Online First: 2014/09/23]
56. Song F, Clark A, Bachmann MO, et al. Simulation evaluation of statistical properties of methods for indirect and mixed treatment comparisons. *BMC medical research methodology* 2012;12:138. doi: 10.1186/1471-2288-12-138 [published Online First: 2012/09/14]
57. Turner RM, Davey J, Clarke MJ, et al. Predicting the extent of heterogeneity in meta-analysis, using empirical data from the Cochrane Database of Systematic Reviews. *Int J Epidemiol* 2012;41(3):818-27. doi: 10.1093/ije/dys041 [published Online First: 2012/03/31]
58. Chaimani A, Higgins JP, Mavridis D, et al. Graphical tools for network meta-analysis in STATA. *PLoS One* 2013;8(10):e76654. doi: 10.1371/journal.pone.0076654 [published Online First: 2013/10/08]
59. Chaimani A, Salanti G. Using network meta-analysis to evaluate the existence of small-study effects in a network of interventions. *Res Synth Methods* 2012;3(2):161-76. doi: 10.1002/jrsm.57 [published Online First: 2012/06/01]
60. Mavridis D, Efthimiou O, Leucht S, et al. Publication bias and small-study effects magnified effectiveness of antipsychotics but their relative ranking remained invariant. *J Clin Epidemiol* 2016;69:161-9. doi: 10.1016/j.jclinepi.2015.05.027 [published Online First: 2015/07/27]
61. Salanti G, Del Giovane C, Chaimani A, et al. Evaluating the quality of evidence from a network meta-analysis. *PLoS One* 2014;9(7):e99682. doi: 10.1371/journal.pone.0099682 [published Online First: 2014/07/06]

For peer review only

STATEMENTS

Declaration

The corresponding author had full access to all the data in the study and the final responsibility for the decision to submit for publication.
The senior author MK (the manuscript's guarantor) affirms that the manuscript is an honest, accurate, and transparent account of the study being reported and that no important aspects of the study have been omitted.

Author statement

The study was conceived and designed by MK, GS and EP. The protocol was drafted by AA, MK, AAV, OE, IK, GS and was revised critically for important intellectual content by all authors (AA, AAV, OE, IK, HN, SB, MP, PMH, PB, EP, GS, MK).

For peer review only - http://bmjopen.bmj.com/site/about/guidelines.xhtml

Acknowledgements
We thank the Jo's Trust for their assistance to involve public and patient representatives in this project.

Funding statement
This work is supported by National Institute for Health Research (NIHR) Research for Patient Benefits (P67307)(MK). MK is also supported by the British Society of Colposcopy Cervical Pathology Jordan/Singer Award (P47773) and the Imperial College Healthcare Charity (P47907)(MK). AAV is also supported by the European Union's Horizon 2020 (No 754936). None of the funders have any influence on the study design; in the collection, analysis, and interpretation of data; in the writing of the report; and in the decision to submit the article for publication.

Competing interests statement
We have read and understood BMJ policy on declaration of interests and declare that we have no conflict of interests. All authors have completed the ICMJE uniform disclosure form at www.icmje.org/coi_disclosure.pdf and declare: no support from any organisation for the submitted work; no financial relationships with any organisations that might have an interest in the submitted work in the previous three years; no other relationships or activities that could appear to have influenced the submitted work.
Dr Kyrgiou has received travel and conference expenses, honoraria and consultancy fees for commercial companies (Inovio, MSD etc); these activities are not related to the project.

Data sharing
No additional data available.

Copyright statement
"The Corresponding Author has the right to grant on behalf of all authors and does grant on behalf of all authors, a worldwide licence to the Publishers and its licensees in perpetuity, in all forms, formats and media (whether known now or created in the future), to i) publish, reproduce, distribute, display and store the Contribution, ii) translate the Contribution into other languages, create adaptations, reprints, include within collections and create summaries, extracts and/or, abstracts of the Contribution, iii) create any other derivative work(s) based on the Contribution, iv) to exploit all subsidiary rights in the Contribution, v) the inclusion of electronic links from the Contribution to third party material where-ever it may be located; and, vi) licence any third party to do any or all of the above."

For peer review only

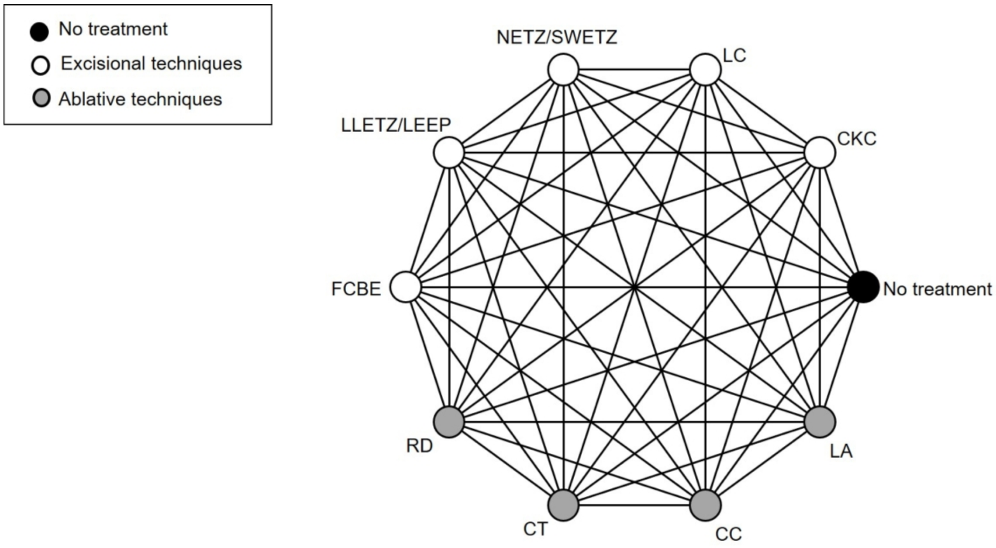

Network of possible pairwise comparisons between eligible treatment methods

203x112mm (300 x 300 DPI)

Supplementary File 1: Reporting checklist for protocol of a systematic review

Based on the PRISMA-P guidelines.

		Reporting Item	Page Number
Identification	#1a	Identify the report as a protocol of a systematic review	1
Update	#1b	If the protocol is for an update of a previous systematic review, identify as such	N/A (no previous NMA)
	#2	If registered, provide the name of the registry (such as PROSPERO) and registration number	2
Contact	#3a	Provide name, institutional affiliation, e-mail address of all protocol authors; provide physical mailing address of corresponding author	1
Contribution	#3b	Describe contributions of protocol authors and identify the guarantor of the review	15
	#4	If the protocol represents an amendment of a previously completed or published protocol, identify as such and list changes; otherwise, state plan for documenting important protocol amendments	4
Sources	#5a	Indicate sources of financial or other support for the review	15
Sponsor	#5b	Provide name for the review funder and / or sponsor	15
Role of sponsor or funder	#5c	Describe roles of funder(s), sponsor(s), and / or institution(s), if any, in developing the protocol	15
Rationale	#6	Describe the rationale for the review in the context of what is already known	3-4
Objectives	#7	Provide an explicit statement of the question(s) the review will address with reference to participants, interventions, comparators, and outcomes (PICO)	4
Eligibility criteria	#8	Specify the study characteristics (such as PICO, study design, setting, time frame) and report characteristics (such as years considered, language, publication status) to be used as criteria for eligibility for the review	4-5
Information sources	#9	Describe all intended information sources (such as electronic	5-6

For peer review only

		databases, contact with study authors, trial registers or other grey literature sources) with planned dates of coverage	
Search strategy	#10	Present draft of search strategy to be used for at least one electronic database, including planned limits, such that it could be repeated	5; Supplementary File 2
Study records - data management	#11a	Describe the mechanism(s) that will be used to manage records and data throughout the review	6
Study records - selection process	#11b	State the process that will be used for selecting studies (such as two independent reviewers) through each phase of the review (that is, screening, eligibility and inclusion in meta-analysis)	6
Study records - data collection process	#11c	Describe planned method of extracting data from reports (such as piloting forms, done independently, in duplicate), any processes for obtaining and confirming data from investigators	6
Data items	#12	List and define all variables for which data will be sought (such as PICO items, funding sources), any pre-planned data assumptions and simplifications	6
Outcomes and prioritization	#13	List and define all outcomes for which data will be sought, including prioritization of main and additional outcomes, with rationale	5
Risk of bias in individual studies	#14	Describe anticipated methods for assessing risk of bias of individual studies, including whether this will be done at the outcome or study level, or both; state how this information will be used in data synthesis	6-7
Data synthesis	#15a	Describe criteria under which study data will be quantitatively synthesised	7-8
	#15b	If data are appropriate for quantitative synthesis, describe planned summary measures, methods of handling data and methods of combining data from studies, including any planned exploration of consistency (such as I2, Kendall's τ)	7-9
	#15c	Describe any proposed additional analyses (such as sensitivity or subgroup analyses, meta-regression)	9
	#15d	If quantitative synthesis is not appropriate, describe the type of summary planned	N/A
Meta-bias(es)	#16	Specify any planned assessment of meta-bias(es) (such as	9

For peer review only

		publication bias across studies, selective reporting within studies)	
Confidence in cumulative evidence	#17	Describe how the strength of the body of evidence will be assessed (such as GRADE)	9

The PRISMA-P checklist is distributed under the terms of the Creative Commons Attribution License CC-BY 4.0. This checklist can be completed online using https://www.goodreports.org/, a tool made by the EQUATOR Network in collaboration with Penelope.ai

For peer review only

Supplementary File 2: Search algorithms

Medline Ovid

1 exp Uterine Cervical Neoplasms/
2 (cervi* and (cancer* or tumor* or tumour* or neoplas* or malignan* or carcinom*)).mp.
3 exp Cervical Intraepithelial Neoplasia/
4 CIN.mp.
5 (cervi* and (intraepithel* or epithel* or dysplasia or pre-cancer* or precancer*)).mp.
6 or/1-5
7 exp Conization/
8 (conisation or conization).mp.
9 exp Laser Therapy/
10 laser.mp.
11 exp Cryotherapy/
12 cryotherapy.mp.
13 cold coagulation.mp.
14 exp Diathermy/
15 diatherm*.mp.
16 cone biopsy.mp.
17 loop.mp.
18 LLETZ.mp.
19 LEEP.mp.
20 ablat*.mp.
21 excision*.mp.
22 transformation zone.mp.
23 (CKC or LA or LC or CC or RD or TZ).mp.
24 (conservative and (method* or treatment* or intervention* or management)).mp.
25 or/7-24
26 6 and 25
27 exp Premature Birth/
28 (preterm or premature).mp.
29 exp Infant, Low Birth Weight/
30 birth weight.mp.
31 Perinatal Mortality/
32 perinatal mortality.mp.
33 exp Intensive Care, Neonatal/
34 (neonatal and intensive care).mp.
35 exp Fertility/
36 fertil*.mp.
37 conception.mp.
38 exp Pregnancy/
39 pregnancy.mp.
40 gestation*.mp.
41 exp Abortion, Spontaneous/
42 miscarriage*.mp.
43 exp Cesarean Section/
44 (cesarean or caesarean).mp.
45 exp Obstetric Labor, Premature/
46 exp Labor, Obstetric/
47 (labor or labour).mp.

For peer review only

48 Fetal Membranes, Premature Rupture/
49 pPROM.mp.
50 or/27-49
51 26 and 50
key:
mp=title, original title, abstract, name of substance word, subject heading word

Embase Ovid
1 exp uterine cervix tumor/
2 (cervi* and (cancer* or tumor* or tumour* or neoplas* or malignan* or carcinom*)).mp.
3 uterine cervix carcinoma in situ/
4 CIN.mp.
5 (cervi* and (intraepithel* or epithel* or dysplasia or pre-cancer* or precancer*)).mp.
6 or/1-5
7 uterine cervix conization/
8 (conisation or conization).mp.
9 low level laser therapy/
10 laser.mp.
11 exp cryotherapy/
12 cryotherapy.mp.
13 cold coagulation.mp.
14 diathermy/
15 diatherm*.mp.
16 cone biopsy.mp.
17 loop.mp.
18 LLETZ.mp.
19 LEEP.mp.
20 ablat*.mp.
21 excision*.mp.
22 transformation zone.mp.
23 (CKC or LA or LC or CC or RD or TZ).mp.
24 (conservative and (method* or treatment* or intervention* or management)).mp.
25 or/7-24
26 6 and 25
27 prematurity/
28 (preterm or premature).mp.
29 exp low birth weight/
30 birth weight.mp.
31 perinatal mortality/
32 perinatal mortality.mp.
33 newborn intensive care/
34 (neonat* and intensive care).mp.
35 female fertility/
36 fertil*.mp.
37 conception/
38 conception.mp.
39 exp pregnancy/
40 pregnancy.mp.
41 gestation*.mp.
42 spontaneous abortion/

For peer review only

43 miscarriage*.mp.
44 cesarean section/
45 (cesarean or caesarean).mp.
46 premature labor/
47 (labor or labour).mp.
48 premature fetus membrane rupture/
49 pPROM.mp.
50 or/27-49
51 26 and 50
key:
mp=title, abstract, subject headings, heading word, drug trade name, original title, device manufacturer, drug manufacturer name

CENTRAL
#1 MeSH descriptor **Uterine Cervical Neoplasms** explode all trees
#2 cervi* and (cancer* or tumor* or tumour* or neoplas* or malignan* or carcinom*)
#3 MeSH descriptor **Cervical Intraepithelial Neoplasia** explode all trees
#4 CIN
#5 cervi* and (intraepithel* or epithel* or dysplasia or pre-cancer* or precancer*)
#6 (#1 OR #2 OR #3 OR #4 OR #5)
#7 MeSH descriptor **Conization** explode all trees
#8 conisation or conization
#9 MeSH descriptor **Laser Therapy** explode all trees
#10 laser
#11 MeSH descriptor **Cryotherapy** explode all trees
#12 cryotherapy
#13 cold coagulation
#14 MeSH descriptor **Diathermy** explode all trees
#15 diatherm*
#16 cone biopsy
#17 loop
#18 LLETZ
#19 LEEP
#20 ablat*
#21 excision*
#22 transformation zone
#23 CKC or LA or LC or CC or RD or TZ
#24 conservative and (method* or treatment* or intervention* or management)
#25 (#7 OR #8 OR #9 OR #10 OR #11 OR #12 OR #13 OR #14 OR #15 OR #16 OR #17 OR #18 OR #19 OR #20 OR #21 OR #22 OR #23 OR #24)
#26 (#6 AND #25)
#27 MeSH descriptor **Premature Birth** explode all trees
#28 preterm or premature
#29 MeSH descriptor **Infant, Low Birth Weight** explode all trees
#30 birth weight
#31 MeSH descriptor **Perinatal Mortality** explode all trees
#32 perinatal mortality
#33 MeSH descriptor **Intensive Care, Neonatal** explode all trees
#34 neonat* and (intensive care)
#35 MeSH descriptor **Fertility** explode all trees
#36 fertil*

For peer review only

#37 conception
#38 MeSH descriptor **Pregnancy** explode all trees
#39 pregnancy
#40 gestation*
#41 MeSH descriptor **Abortion, Spontaneous** explode all trees
#42 miscarriage*
#43 MeSH descriptor **Cesarean Section** explode all trees
#44 cesarean or caesarean
#45 MeSH descriptor **Obstetric Labor, Premature** explode all trees
#46 MeSH descriptor **Labor, Obstetric** explode all trees
#47 labor or labour
#48 MeSH descriptor **Fetal Membranes, Premature Rupture** explode all trees
#49 pPROM
#50 (#27 OR #28 OR #29 OR #30 OR #31 OR #32 OR #33 OR #34 OR #35 OR #36 OR #37 OR #38 OR #39 OR #40 OR #41 OR #42 OR #43 OR #44 OR #45 OR #46 OR #47 OR #48 OR #49)
#51 (#26 AND #50)

For peer review only

BMJ Open

Comparative fertility and pregnancy outcomes after local treatment for cervical intra-epithelial neoplasia and stage 1a1 cervical cancer: protocol for a systematic review and network meta-analysis from the CIRCLE Group

Journal:	*BMJ Open*
Manuscript ID	bmjopen-2018-028009.R2
Article Type:	Protocol
Date Submitted by the Author:	11-Sep-2019
Complete List of Authors:	Athanasiou, Antonios; Institute of Reproductive and Developmental Biology, Department of Surgery & Cancer, Faculty of Medicine, Imperial College London; Imperial College Healthcare NHS Trust<br>Veroniki, Areti Angeliki; Institute of Reproductive and Developmental Biology, Department of Surgery & Cancer, Faculty of Medicine, Imperial College London; Panepistimio Ioanninon, School of Education, Department of Primary Education<br>Efthimiou, Orestis; University of Bern, Institute of Social and Preventive Medicine (ISPM)<br>Kalliala, Ilkka; Institute of Reproductive and Developmental Biology, Department of Surgery & Cancer, Faculty of Medicine, Imperial College London; University of Helsinki and Helsinki University Hospital, Department of Obstetrics and Gynaecology<br>Naci, Huseyin; London School of Economics, Department of Health Policy<br>Bowden, Sarah; Institute of Reproductive and Developmental Biology, Department of Surgery & Cancer, Faculty of Medicine, Imperial College London; Imperial College Healthcare NHS Trust<br>Paraskevaidi, Maria; Institute of Reproductive and Developmental Biology, Department of Surgery & Cancer, Faculty of Medicine, Imperial College London<br>Martin-Hirsch, Pierre; Lancashire Teaching Hospitals NHS Foundation Trust, Department of Gynaecologic Oncology<br>Bennett, Philip; Institute of Reproductive and Developmental Biology, Department of Surgery & Cancer, Faculty of Medicine, Imperial College London; Imperial College Healthcare NHS Trust<br>Paraskevaidis, Evangelos; University of Ioannina and University Hospital of Ioannina, Department of Obstetrics & Gynaecology; Imperial College Healthcare NHS Trust<br>Salanti, Georgia; University of Bern, Institute of Social and Preventive Medicine (ISPM)<br>Kyrgiou, Maria; Institute of Reproductive and Developmental Biology, Department of Surgery & Cancer, Faculty of Medicine, Imperial College London; Imperial College Healthcare NHS Trust
**Primary Subject Heading**:	Obstetrics and gynaecology
Secondary Subject Heading:	Oncology, Evidence based practice

Keywords:	"Cervical Intraepithelial Neoplasia/surgery"[Mesh], "Cervical Intraepithelial Neoplasia/therapy"[Mesh], "Conization/adverse effects"[Mesh], "Ablation Techniques/adverse effects"[Mesh], "Pregnancy/complications"[Mesh], "Premature Birth"[Mesh

SCHOLARONE™
Manuscripts

Comparative fertility and pregnancy outcomes after local treatment for cervical intra-epithelial neoplasia and stage 1a1 cervical cancer: protocol for a systematic review and network meta-analysis from the CIRCLE Group

Antonios Athanasiou[1,2], Areti Angeliki Veroniki[1,3], Orestis Efthimiou[4], Ilkka Kalliala[1,5], Huseyin Naci[6], Sarah Bowden[1,2], Maria Paraskevaidi[1], Pierre Martin-Hirsch[7], Phillip Bennett[1,2], Evangelos Paraskevaidis[2,8], Georgia Salanti[4], Maria Kyrgiou[1,2]

[1]Institute of Reproductive and Developmental Biology, Department of Surgery & Cancer, Faculty of Medicine, Imperial College London, UK
[2]Imperial Healthcare NHS Trust, London, UK
[3]Department of Primary Education, School of Education, University of Ioannina, Greece
[4]Institute of Social and Preventive Medicine (ISPM), University of Bern, Mittelstrasse 43, 3012 Bern, Switzerland
[5]Department of Obstetrics and Gynaecology, University of Helsinki and Helsinki University Hospital, Finland
[6]Department of Health Policy, London School of Economics & Political Science, UK
[7]Department of Gynaecologic Oncology, Lancashire Teaching Hospitals, Preston, UK
[8]Department of Obstetrics & Gynaecology, University of Ioannina and University Hospital of Ioannina, Greece

AA: a.athanasiou@imperial.ac.uk; AAV: a.veroniki@imperial.ac.uk; OE: oremiou@gmail.com; IK: i.kalliala@imperial.ac.uk; HN: h.naci@lse.ac.uk; SB: s.lever@imperial.ac.uk; MP: m.paraskevaidi@imperial.ac.uk; PMH: pierre.martin-hirsch@lthtr.nhs.uk; PB: p.bennett@imperial.ac.uk; EP: eparaske@cc.uoi.gr; GS: georgia.salanti@ispm.unibe.ch; MK: m.kyrgiou@imperial.ac.uk

Corresponding author:
Maria Kyrgiou, MSc, PhD, MRCOG
3rd Floor, IRDB, Department of Surgery & Cancer
Imperial College London, Hammersmith Campus
Du Cane Road, W12 0NN, London
Email: m.kyrgiou@imperial.ac.uk - Tel: +44 2075942177

Word Count:

KEYWORDS
"Cervical Intraepithelial Neoplasia/surgery"[Mesh]
"Cervical Intraepithelial Neoplasia/therapy"[Mesh]
"Conization/adverse effects"[Mesh]
"Ablation Techniques/adverse effects"[Mesh]
"Pregnancy/complications"[Mesh]
"Premature Birth"[Mesh]

For peer review only

ABSTRACT

Introduction: There are several local treatment methods for cervical intra-epithelial neoplasia that remove or ablate a cone-shaped part of the uterine cervix. There is evidence to suggest that these increase the risk of preterm birth and that this is higher for techniques that remove larger parts of the cervix, although the data is conflicting. We present a protocol for a systematic review and network meta-analysis that will update the evidence and compare all treatments in terms of fertility and pregnancy complications.

Methods and Analysis: We will search electronic databases (CENTRAL, MEDLINE, EMBASE) from inception till October 2019, in order to identify randomised controlled trials (RCTs) and cohort studies comparing the fertility and pregnancy outcomes amongst different excisional and ablative treatment techniques and/or to untreated controls. The primary outcome will be preterm birth (PTB; <37weeks). Secondary outcomes will include severe or extreme PTB, prelabour rupture of membranes, low birth weight (<2500gr), neonatal intensive care unit admission, perinatal mortality, total pregnancy rates, 1st and 2nd trimester miscarriage. We will search for published and unpublished studies in electronic databases, trial registries and we will hand-search references of published papers. We will assess the risk of bias in RCTs and cohort studies using tools developed by the Cochrane Collaboration. Two investigators will independently assess the eligibility, abstract the data and assess the risk of bias of the identified studies. For each outcome, we will perform a meta-analysis for each treatment comparison and a network meta-analysis once the transitivity assumption holds, using the odds ratio for dichotomous data. We will use CINEMA to assess the quality of the evidence for the primary outcome.

Ethics and dissemination: Ethical approval is not required. Results will be disseminated to academic beneficiaries, medical practitioners, patients and the public.

PROSPERO registration number: CRD42018115495

ARTICLE SUMMARY

Strengths and limitations of this study

- This will be the first network meta-analysis to produce comprehensive summaries of the relative reproductive morbidity of treatment methods for cervical pre-invasive and early invasive disease.
- We will use state-of-the-art methods for combining randomised and non-randomised studies in a network meta-analysis.
- Risk of bias will be evaluated at both study and outcome level.
- One possible limitation of this review is that we expect to find mainly retrospective cohort studies at high risk of recall, selection and publication bias.

For peer review only

INTRODUCTION

The introduction of systematic call and recall screening programmes in the UK has resulted in a profound decrease in the incidence and mortality from cervical cancer, as pre-invasive precursors (cervical intra-epithelial neoplasia; CIN) can be detected by the screening programme and treated[1]. In England alone, 3.6 million women aged 25 to 64 attended for screening in 2013-14 and over 23,800 treatment procedures were carried out[2].

Local conservative treatment for cervical pre-invasive and early invasive disease removes or ablates a cone-shaped part of the cervix containing the precancerous cells. The choice of technique varies within the UK, across Europe and beyond. In some countries, knife excision (cold knife conisation; CKC) is still regularly performed; in others, ablation (LA) or conisation (LC) with the laser beam is common practice. In the UK, large loop excision of the transformation zone (LLETZ) is the preferred treatment, with some units offering alternative techniques more frequently than others. This preference is because LLETZ is quick, easy to do and of low cost.

The mean age of women undergoing CIN treatment is similar to the age of women having their first child. Although, previously, complications from treatment were thought to be relatively mild and uncommon, an increasing body of retrospective observational studies and meta-analyses suggested that treatment, particularly excision, adversely affects future reproduction and the risk of prematurity[3-8]. It has been suggested that the frequency and severity of the observed adverse events is higher for the more radical techniques and with increasing cone depth[4 7 9-14]. Preterm birth (PTB) is a major cause of neonatal death and disability and represents an enormous cost to the health services and the society in general.

Although all treatment techniques are highly effective in preventing recurrent pre-cancerous disease and future invasion[15], some of the data on the risk of reproductive morbidity for the treatment methods has been conflicting[3 5 7 10 16-18] and their comparative reproductive morbidity remains unclear. With some authors raising concerns that the progressive reduction in the radicality of treatment has led to increased risk of future post-treatment invasive disease[19 20], and others advocating the move to less radical techniques, such as laser ablation, for the prevention of treatment-associated adverse obstetric outcomes, such as preterm birth or perinatal mortality[4 21], high-quality synthesis of the current evidence base is an urgent unmet need. Given the premalignant nature of the condition, an RCT comparing the various treatment technique to no treatment will never be conducted.

The quantification of the comparative reproductive morbidity of different treatment techniques and cone lengths has become a women's health priority. This requires high-quality synthesis of the evidence in comprehensive summaries that will become available for effective patient counselling at colposcopy and antenatal clinics for patients, clinicians and policy makers. A clinical ranking of treatments with regards to the risk of preterm birth may allow the quantification of risk and the detection of women at high risk of PTB that would benefit from intensive surveillance antenatally, while minimising the unnecessary interventions for those at lower risk.

For peer review only - http://bmjopen.bmj.com/site/about/guidelines.xhtml

The key methodological vehicle to synthesize evidence is systematic reviews and their quantitative component, meta-analysis. Network meta-analysis (NMA) is an extension of pairwise meta-analysis, which can be used to estimate the relative effectiveness of several competing treatments. An NMA has never been used before to assess the comparative efficacy and harms of the different treatment techniques in this field. For every treatment comparison NMA synthesizes both direct evidence (i.e. coming from studies comparing head-to-head the treatments of interest) and indirect evidence (i.e. coming from studies comparing the treatments of interest via an intermediate common comparator)[22-25]. In addition, NMA allows the estimation of relative effects between all available treatments, can lead to an increased precision as compared to the pairwise meta-analysis and provides a ranking of the available competing treatments. The potential of NMA has been recognized by the National Institute of Clinical Excellence (NICE)[26] and several international Health Technology Assessment (HTA) agencies[27 28].

The objective of this systematic review and NMA is to update the evidence and to compare the various local treatment methods to manage CIN in terms of fertility, early (<24 weeks of gestation) and late (>24 weeks) pregnancy complications. This is part of the CIRCLE project (**C**ervical Cancer Incidence, C**I**N Recurrence and **R**eproduction after **L**ocal **E**xcision), which aims to generate a clinically useful raking of alternative options for treatment of CIN according to their efficacy (risk of pre-invasive and invasive recurrence), morbidity and cost-effectiveness.

METHODS AND ANALYSIS

This protocol complies with the Preferred Reporting Items for Systematic Review and Meta-Analysis Protocols (PRISMA-P) (Supplementary File 1)[29]. Any changes in this protocol will be recorded in an updated version of the PROSPERO registration.

Criteria for considering studies for this review

Types of participants

We will include women of all ages with a prior history of local surgical treatment for CIN or micro-invasive early cervical cancer (stage IA1). Their status can be confirmed with histological or cytological diagnosis and irrespective of the grade of the treated lesion for both squamous and glandular intra-epithelial neoplasia. Studies recruiting solely women at high risk of preterm birth (such as previous history of preterm birth) or studies including only patients treated during pregnancy will be excluded.

Types of interventions

We aim to compare 9 different excisional or ablative techniques used for conservative treatment for cervical intraepithelial neoplasia (CIN). The excisional techniques include cold knife conisation (CKC); laser conisation (LC); needle excision of the transformation zone (NETZ), also known as straight wire excision of the transformation zone (SWETZ); large loop excision of the transformation zone (LLETZ), also known as loop electrosurgical

For peer review only - http://bmjopen.bmj.com/site/about/guidelines.xhtml

excisional procedure (LEEP); Fischer cone biopsy excision (FCBE). The ablative techniques include radical point diathermy (RD); cryotherapy (CT); cold coagulation (CC); laser ablation (LA). We will include studies comparing any of these treatments with each other or with no treatment. Figure 1 shows a network example of all possible comparisons between eligible interventions for the primary analysis. If the specific treatment technique is not specified, these will be grouped under the broader categories excision (ENS) or ablation (ANS).

Outcome measures

Primary outcome

- PTB, defined as less than 37 weeks of gestation

Secondary outcomes

- Spontaneous PTB (sPTB), defined as less than 37 weeks of gestation
- Severe PTB, defined as less than 32/34 weeks of gestation
- Extreme PTB, defined as less than 28/30 weeks of gestation
- PROM (Prelabour Rupture of Membranes) defined as membrane rupture before the onset of labour
- Low Birth Weight (LBW), defined as infant born weighing less than 2500g (late neonatal obstetric outcome)
- Neonatal Intensive Care Unit (NICU) admission
- Perinatal mortality, defined as the number of stillbirths and neonatal deaths occurring within 28 days after birth
- Total pregnancy rate, defined as any pregnancy occurring from CIN treatment till study completion irrespective of outcome (miscarriage, ectopic, molar pregnancy, termination of pregnancy, live birth, stillbirth)'.
- Rates of women requiring more than 12 months to conceive
- 1st trimester miscarriage, defined as miscarriage at less than 12 weeks of gestation
- 2nd trimester miscarriage, defined as miscarriage between 12-24 weeks of gestation

Outcomes and their classification into primary or secondary were decided after clinical experts' opinion. Total pregnancy rate will be recorded for the whole study period and/or prespecified intervals, if data is available.

Types of studies

We will include randomised controlled trials (RCTs), quasi-RCTs and cohort studies comparing fertility and early (<24 weeks of gestation) or late (>24 weeks of gestation) pregnancy outcomes amongst surgical techniques and those that compare to no treatment. Studies that do not perform a comparison between treatments (i.e. "single-arm studies") will be excluded. Studies that compared a treatment with an untreated group will be included irrespective of the type of the untreated group (e.g. studies that used data from untreated women from the general population; studies that used self-matching, i.e. including women with pregnancies before and after treatment; studies that used data from women with a

For peer review only - http://bmjopen.bmj.com/site/about/guidelines.xhtml

history of abnormal cytology/HPV infection/untreated CIN). There will be no time or language restriction.

Information sources and search strategy

An experienced librarian will search The Cochrane Gynaecological Cancer Specialised Register; Cochrane Central Register of Controlled Trials (CENTRAL); MEDLINE; and EMBASE for eligible studies from inception. The search algorithms for these databases are presented in Supplementary File 2. We will search Metaregister, Physicians Data Query, www.controlled-trials.com/rct, www.clinicaltrials.gov and www.cancer.gov/clinicaltrials for ongoing studies. There will be no time or language restriction.

We will contact the corresponding author of any relevant ongoing trials for further information and unpublished data. In an attempt to identify any articles missed by the initial search, we will use the 'related articles' feature in MEDLINE. We will also hand search the references of the retrieved articles and meta-analyses. We will search conference proceedings and abstracts through ZETOC (http://zetoc.mimas.ac.uk), and theses through WorldCat Dissertations. The selected conferences will include: British Society of Colposcopy and Cervical Pathology; International Federation of Cervical Pathology and Colposcopy; Annual Meeting of European Federation of Colposcopy; Annual Meeting of the American Society of Colposcopy and Cervical Pathology. We will contact experts in the field, including directors of UK cancer and colposcopy registries, to identify further reports of studies. We will include both published and unpublished studies.

Study selection

We will download abstracts retrieved into a reference management software, Zotero. Then, two persons will independently review titles and abstracts retrieved by the search (level 1). At level 2, we will obtain the full text of all included articles and two reviewers will independently use the same inclusion criteria to determine eligibility. Disagreements at any level will be resolved via discussion with a third member of the review team.

Data collection

Two reviewers will extract data independently using a standardised data collection form in Excel. Disagreements will be resolved through discussion. Information extracted will include study characteristics (such as author, publication year and study design), participants (such as age, CIN grade and smoking) and comparison group characteristics, setting, inclusion/exclusion criteria, intervention details, outcome measures and dropout rates. In RCTs we will prefer arm-level data (number of events and sample size per intervention arm), but if these are missing, the study-level data will be used in the analysis, e.g. reported odds ratios (ORs) and a measure of their uncertainty (e.g. confidence interval). In observational studies we will extract estimates of treatment effects that are adjusted for the lack of randomisation, i.e. after taking into account the impact of potential confounders, or, if these are missing, the reported unadjusted estimates, as well as the corresponding uncertainty measure.

For peer review only - http://bmjopen.bmj.com/site/about/guidelines.xhtml

Risk of bias assessment

Results of the meta-analyses will be interpreted in light of risk of bias assessment of the included studies. Two investigators will independently assess the methodological quality/risk of bias of the studies that fulfil the eligibility criteria and differences will be resolved by discussion with a third investigator.

For RCTs, the risk of bias will be assessed using the tool[30] developed by Cochrane assessing the following domains: randomisation process, deviations from the intended interventions, missing outcome data, measurement of the outcome and selection of the reported result. The risk of bias in each domain, as well as the overall risk of bias, will be rated as "low risk", "some concerns", or "high risk", after answering the signalling questions of each domain with "Yes", "Potentially Yes", "Potentially No" or "No". When inadequate details are reported in the study to be able to rate a risk of bias item, we will contact the study authors for additional information.

For non-randomised studies (NRS), we will use the ROBINS-I tool[31] developed by the Cochrane collaboration that facilitates the evaluation of the risk of bias by considering that each NRS is an attempt to mimic an RCT comparing the effects of the intervention or exposure studied. During the review stage, we will evaluate the risk of bias in the following domains: confounding, selection of participants into the study, classification of interventions, deviations from intended interventions, missing data, measurement of outcomes and selection of the reported results. The confounding factors that we will evaluate are age, parity and smoking. Each ROBINS-I domain and the overall risk of bias will be assessed as "low", "moderate", "serious", or "critical", after answering the signalling questions of each domain with "Yes", "Potentially Yes", "Potentially No" or "No".

Statistical synthesis

Characteristics of included studies and Network

We will generate descriptive statistics for eligible studies and study population characteristics, describing the types of comparisons and important clinical or methodological variables (such as publication year, study design and source of data). We will present the evidence in a network diagram per outcome. The total number of patients will be reflected in the size of the nodes, while the weight of each edge will be proportional to the number of studies per treatment comparison.

Pairwise meta-analyses

We will synthesise data to obtain summary ORs for dichotomous outcomes with a 95% CI in an inverse variance random-effects model assuming that the studies are estimating different but related treatment effects. In each meta-analysis, we will estimate the between-study variance with the restricted maximum likelihood estimator[32 33] and its 95% CI using the Q-profile approach[34]. We will assess between-study variance using the I^2 statistic along a 95% confidence interval (CI)[35 36]. We will estimate each summary effect size and its 95% CI using the Hartung-Knapp-Sidik-Jonkman method[32 37 38] to handle meta-analyses that include a

For peer review only - http://bmjopen.bmj.com/site/about/guidelines.xhtml

small number of studies. All meta-analyses will be conducted in R[39] using the *metafor* package[40].

Network meta-analyses (NMA)

Data synthesis

We will fit a random effects NMA model, taking into account the correlation induced by multi-arm studies[41]. We will use a random-effects model, since we anticipate methodological and clinical heterogeneity across studies. We will assume a common between-study variance parameter for all comparisons in the network, so that treatment comparisons informed by a single study can borrow strength from the remaining network[42 43]. Clinically, this assumption is reasonable because all treatments included in the network are of the same nature. We will estimate the common between-study variance with the DerSimonian and Laird method of moments approach[44].

We expect that we will include several NRS in our dataset. In that case, we will employ the methods described by Efthimiou et al.[45], i.e. a "design-adjusted", and a three-level hierarchical NMA model. Using these methods, we will incorporate the totality of available information, both randomized and observational, in a joint NMA. We will start by analysing the study-specific estimates from the NRS at face value. Then, in extensive sensitivity analyses we will explore the impact of assigning different levels of credibility and subsequently down-weighting the NRS, according to experts' opinion and the risk of bias as assessed in ROBINS-I.

For each treatment comparison we will report the estimated OR, the 95% CI and the 95% prediction intervals. We will also estimate the ranking probabilities for all treatments of being at each possible rank for each intervention. Thus, we will obtain a treatment hierarchy using the surface under the cumulative ranking curve (SUCRA) or P-scores and mean ranks[46]. A rank-heat plot will be used to depict the SUCRA values or P-scores for all outcomes[47 48]. All NMAs will be fit in R[39] with the *netmeta*[49] and *rjags*[50] package.

We will repeat our NMAs after grouping all excisional and all ablative techniques together; see groupings of treatments in Figure 1.

Assessment of the transitivity assumption

NMA rests on the assumption of transitivity, i.e. that effect modifiers have a similar distribution across treatment comparisons in a network[24 51 52]. In order to assess the plausibility of this assumption, we will summarize study and patient-level characteristics that are expected to influence relative treatment effects, for each pairwise comparison for which direct evidence is available in the network. In this NMA, the most important effect modifiers are expected to be year of study, method of ascertainment of exposure/outcome (hospital records, registries or interviews/questionnaires), age, parity, smoking and CIN grade. We will visually inspect the similarity of the identified studies in terms of these effect modifiers. We will investigate the inclusion and exclusion criteria of all studies, to make sure that patients,

For peer review only - http://bmjopen.bmj.com/site/about/guidelines.xhtml

treatments and outcomes in the studies are sufficiently similar in all aspects that are expected to modify relative treatment effects.

Assessment of statistical inconsistency

Checking the network for inconsistency offers an additional way of assessing the validity of the transitivity assumption. In order to evaluate the presence of inconsistency locally, we will separate the indirect from the direct evidence for each comparison and infer about their differences following the back-calculation method[53]. We will also follow a global approach for assessing consistency in the network, by applying the design-by-treatment interaction model[54]. Simulations suggest that inconsistency tests have low power to detect true inconsistency[55 56]. Therefore, we will conceptually assess the transitivity assumption (see previous paragraph) even in the absence of evidence for inconsistency. We will perform both local (back-calculation method) and global (design-by-treatment interaction model) assessments in R[39] using the *netmeta* package[49].

When a network includes both randomised and non-randomised evidence, we will also explore differences between the different types of evidence, as discussed in Efthimiou et al[45]. For each treatment comparison there may be up to four different types of evidence: direct randomised, indirect randomised, direct non-randomised and indirect non-randomised. We will summarise all evidence by type, for each treatment comparison. If data permits, important discrepancies between these types will be further investigated, as they might indicate a breach of the transitivity assumption (e.g. when randomised and non-randomised evidence are very different in terms of populations, interventions, etc.), or the presence of important, unaccounted confounding in the non-randomised evidence. If a source of disagreement is identified, it will be included in our analysis through network meta-regression models[23].

Exploring heterogeneity and inconsistency: subgroup analyses, meta-regression, and sensitivity analyses

We will assess the extent of statistical heterogeneity by inspecting the 95% prediction intervals and by comparing the estimated value of the between-study variance with the empirical distribution derived by Turner et al for dichotomous data[57]. For the primary outcome, we will explore the following possible sources of heterogeneity and inconsistency: year of study, method of ascertainment of exposure/outcome (hospital records, registries or interviews/questionnaires), age, parity, smoking and CIN grade. If sufficient studies are available, the role of these variables will be explored by means of subgroup analyses (categorical characteristics) or network meta-regressions (continuous characteristics).

Reporting bias and small-study effects

In order to assess possible existence of small studies giving different effect estimates than larger studies, we will visually explore the funnel-plots for each treatment against the untreated group (using the relevant studies) when at least 10 studies inform the underling treatment comparison. We will also assess for small-study effects using the comparison

For peer review only - http://bmjopen.bmj.com/site/about/guidelines.xhtml

adjusted funnel plot[58] and will conduct a network meta-regression using the study variance as a covariate[59 60].

Assessment of the credibility of the evidence

We will evaluate the credibility of the evidence contributing to the network estimates in the primary outcome using CINEMA[61] (http://cinema.ispm.ch/). Two team members will determine the degree of confidence in the estimated NMA results by assessing the six CINEMA domains: within-study bias (i.e., risk of bias in the included studies), across-study bias (i.e., publication and reporting bias), indirectness, imprecision, heterogeneity and incoherence (i.e., differences between direct and indirect evidence)[61]. Each network summary estimate will initially be judged as high quality, but it will be downgraded if this is judged appropriate according to the six domains. Judgements within each domain will be summarized for each NMA relative treatment effect as: very low, low, moderate or high.

Patient and Public involvement

We have discussed the project with Jo's Cervical Cancer Trust (a UK charity who supports patients affected by cervical pre-invasive or invasive cervical disease and campaigns for excellence in cervical cancer treatment and prevention).

Two patient representatives through Jo's Trust with personal experience of cervical disease have assisted us in the design of the study and the development of research questions. We aim to recruit more patients through Jo's Voice, who will help us understand the key priorities from patients' perspective, produce lay summaries and disseminate the results to the wider public.

ETHICS AND DISSEMINATION

This review does not require ethical approval. We identified four groups of potential stakeholders (academic beneficiaries; health-related agencies and decision-makers; medical practitioners; patients and public) and specific action items to effectively target them. We will publish papers in influential open access journals and we will present data at high-profile conferences. We will make the datasets available to the wider research community. We will organise a workshop with key stakeholders. We will develop information sheets and briefings, highlighting the key findings and circulate newsletters. We will engage the press with presentations and social media interviews and we will work closely with Jo's Trust charity that plays an important role in educating patient communities.

Figure 1. Network of possible pairwise comparisons between eligible treatment methods
Abbreviations: **FCBE**=Fischer cone biopsy excision; **LLETZ**= large loop excision of the transformation zone, also known as **LEEP**=loop electrosurgical excisional procedure; **NETZ**=needle excision of the transformation, also known as **SWETZ**=straight wire excision of the transformation zone; **LC**=laser conisation; **CKC**=cold knife conisation; **LA**=laser ablation; **CC**=cold coagulation; **CT**=cryotherapy; **RD**=radical point diathermy

For peer review only - http://bmjopen.bmj.com/site/about/guidelines.xhtml

REFERENCES

1. Quinn M, Babb P, Jones J, et al. Effect of screening on incidence of and mortality from cancer of cervix in England: evaluation based on routinely collected statistics. *BMJ* 1999;318(7188):904-8.
2. PublicHealthEngland. Cervical screening: programme overview. http://www.cancerscreening.nhs.uk/cervical, 2015.
3. Kyrgiou M, Koliopoulos G, Martin-Hirsch P, et al. Obstetric outcomes after conservative treatment for intraepithelial or early invasive cervical lesions: systematic review and meta-analysis. *Lancet* 2006;367(9509):489-98. doi: 10.1016/s0140-6736(06)68181-6 [published Online First: 2006/02/14]
4. Arbyn M, Kyrgiou M, Simoens C, et al. Perinatal mortality and other severe adverse pregnancy outcomes associated with treatment of cervical intraepithelial neoplasia: meta-analysis. *BMJ* 2008;337:a1284. doi: 10.1136/bmj.a1284 [published Online First: 2008/09/20]
5. Kyrgiou M, Mitra A, Arbyn M, et al. Fertility and early pregnancy outcomes after treatment for cervical intraepithelial neoplasia: systematic review and meta-analysis. *Bmj* 2014;349:g6192. doi: 10.1136/bmj.g6192 [published Online First: 2014/10/30]
6. Kyrgiou M, Mitra A, Arbyn M, et al. Fertility and early pregnancy outcomes after conservative treatment for cervical intraepithelial neoplasia. *Cochrane Database Syst Rev* 2015(9):Cd008478. doi: 10.1002/14651858.CD008478.pub2 [published Online First: 2015/09/30]
7. Kyrgiou M, Athanasiou A, Paraskevaidi M, et al. Adverse obstetric outcomes after local treatment for cervical preinvasive and early invasive disease according to cone depth: systematic review and meta-analysis. *Bmj* 2016;354:i3633. doi: 10.1136/bmj.i3633 [published Online First: 2016/07/30]
8. Kyrgiou M, Athanasiou A, Kalliala IEJ, et al. Obstetric outcomes after conservative treatment for cervical intraepithelial lesions and early invasive disease. *Cochrane Database Syst Rev* 2017;11:Cd012847. doi: 10.1002/14651858.Cd012847 [published Online First: 2017/11/03]
9. Kyrgiou M, Arbyn M, Martin-Hirsch P, et al. Increased risk of preterm birth after treatment for CIN. *Bmj* 2012;345:e5847. doi: 10.1136/bmj.e5847 [published Online First: 2012/09/07]
10. Castanon A, Landy R, Brocklehurst P, et al. Risk of preterm delivery with increasing depth of excision for cervical intraepithelial neoplasia in England: nested case-control study. *BMJ* 2014;349:g6223. [published Online First: 2014/11/05]
11. Noehr B, Jensen A, Frederiksen K, et al. Depth of cervical cone removed by loop electrosurgical excision procedure and subsequent risk of spontaneous preterm delivery. *Obstet Gynecol* 2009;114(6):1232-8. doi: 10.1097/AOG.0b013e3181bf1ef2 [published Online First: 2009/11/26]
12. Khalid S, Dimitriou E, Conroy R, et al. The thickness and volume of LLETZ specimens can predict the relative risk of pregnancy-related morbidity. *Bjog* 2012;119(6):685-91. doi: 10.1111/j.1471-0528.2011.03252.x [published Online First: 2012/02/15]

For peer review only - http://bmjopen.bmj.com/site/about/guidelines.xhtml

13. Founta C, Arbyn M, Valasoulis G, et al. Proportion of excision and cervical healing after large loop excision of the transformation zone for cervical intraepithelial neoplasia. *Bjog* 2010;117(12):1468-74. doi: 10.1111/j.1471-0528.2010.02709.x [published Online First: 2010/09/16]
14. Kyrgiou M, Valasoulis G, Stasinou SM, et al. Proportion of cervical excision for cervical intraepithelial neoplasia as a predictor of pregnancy outcomes. *Int J Gynaecol Obstet* 2015;128(2):141-7. doi: 10.1016/j.ijgo.2014.07.038 [published Online First: 2014/12/03]
15. Martin-Hirsch PP, Paraskevaidis E, Bryant A, et al. Surgery for cervical intraepithelial neoplasia. *Cochrane Database Syst Rev* 2013(12):Cd001318. doi: 10.1002/14651858.CD001318.pub3 [published Online First: 2013/12/05]
16. Conner SN, Frey HA, Cahill AG, et al. Loop electrosurgical excision procedure and risk of preterm birth: a systematic review and meta-analysis. *Obstet Gynecol* 2014;123(4):752-61. doi: 10.1097/AOG.0000000000000174 [published Online First: 2014/05/03]
17. Danhof NA, Kamphuis EI, Limpens J, et al. The risk of preterm birth of treated versus untreated cervical intraepithelial neoplasia (CIN): a systematic review and meta-analysis. *Eur J Obstet Gynecol Reprod Biol* 2015;188:24-33. doi: 10.1016/j.ejogrb.2015.02.033 [published Online First: 2015/03/17]
18. Jin G, LanLan Z, Li C, et al. Pregnancy outcome following loop electrosurgical excision procedure (LEEP) a systematic review and meta-analysis. *Arch Gynecol Obstet* 2014;289(1):85-99. doi: 10.1007/s00404-013-2955-0 [published Online First: 2013/07/12]
19. Strander B, Hällgren J, Sparén P. Effect of ageing on cervical or vaginal cancer in Swedish women previously treated for cervical intraepithelial neoplasia grade 3: population based cohort study of long term incidence and mortality. *BMJ* 2014;348:f7361. [published Online First: 2014/01/14]
20. Arbyn M, Kyrgiou M, Gondry J, et al. Long term outcomes for women treated for cervical precancer. *Bmj* 2014;348:f7700. doi: 10.1136/bmj.f7700 [published Online First: 2014/01/16]
21. Paraskevaidis E, Kyrgiou M, Martin-Hirsch P. Have we dismissed ablative treatment too soon in colposcopy practice? *Bjog* 2007;114(1):3-4. doi: 10.1111/j.1471-0528.2006.01178.x [published Online First: 2007/01/20]
22. Caldwell DM, Ades AE, Higgins JP. Simultaneous comparison of multiple treatments: combining direct and indirect evidence. *BMJ* 2005;331(7521):897-900. doi: 10.1136/bmj.331.7521.897
23. Salanti G, Marinho V, Higgins JP. A case study of multiple-treatments meta-analysis demonstrates that covariates should be considered. *J Clin Epidemiol* 2009;62(8):857-64. doi: 10.1016/j.jclinepi.2008.10.001 [published Online First: 2009/01/20]
24. Cipriani A, Higgins JP, Geddes JR, et al. Conceptual and technical challenges in network meta-analysis. *Ann Intern Med* 2013;159(2):130-7. doi: 10.7326/0003-4819-159-2-201307160-00008

For peer review only

For peer review only - http://bmjopen.bmj.com/site/about/guidelines.xhtml

25. Efthimiou O, Debray TP, van Valkenhoef G, et al. GetReal in network meta-analysis: a review of the methodology. *Res Synth Methods* 2016;7(3):236-63. doi: 10.1002/jrsm.1195 [published Online First: 2016/01/13]
26. NICE. Developing NICE guidelines: the manual. Process and methods (PMG20). Reviewing research evidence 2017 [Available from: https://www.nice.org.uk/process/pmg20/chapter/reviewing-research-evidence.
27. Caldwell DM, Dias S, Welton NJ. Extending Treatment Networks in Health Technology Assessment: How Far Should We Go? *Value Health* 2015;18(5):673-81. doi: 10.1016/j.jval.2015.03.1792 [published Online First: 2015/08/25]
28. CADTH. Network Meta-Analysis 2015 [Available from: https://www.cadth.ca/network-meta-analysis.
29. Moher D, Shamseer L, Clarke M, et al. Preferred reporting items for systematic review and meta-analysis protocols (PRISMA-P) 2015 statement. *Syst Rev* 2015;4:1. doi: 10.1186/2046-4053-4-1 [published Online First: 2015/01/03]
30. Higgins JPT, Sterne JAC, Savović J, et al. A revised tool for assessing risk of bias in randomized trials. In: Chandler J, McKenzie J, Boutron I, et al., eds.: Cochrane Methods. Cochrane Database of Systematic Reviews 2016.
31. Sterne JA, Hernan MA, Reeves BC, et al. ROBINS-I: a tool for assessing risk of bias in non-randomised studies of interventions. *BMJ* 2016;355:i4919. doi: 10.1136/bmj.i4919 [published Online First: 2016/10/14]
32. Veroniki AA, Jackson D, Viechtbauer W, et al. Methods to estimate the between-study variance and its uncertainty in meta-analysis. *Res Synth Methods* 2016;7(1):55-79. doi: 10.1002/jrsm.1164 [published Online First: 2015/09/04]
33. Langan D, Higgins JPT, Jackson D, et al. A comparison of heterogeneity variance estimators in simulated random-effects meta-analyses. *Res Synth Methods* 2018 doi: 10.1002/jrsm.1316 [published Online First: 2018/08/02]
34. Viechtbauer W. Confidence intervals for the amount of heterogeneity in meta-analysis. *Stat Med* 2007;26(1):37-52. doi: 10.1002/sim.2514 [published Online First: 2006/02/08]
35. Higgins JP, Thompson SG, Deeks JJ, et al. Measuring inconsistency in meta-analyses. *BMJ* 2003;327(7414):557-60. doi: 10.1136/bmj.327.7414.557 [published Online First: 2003/09/06]
36. Veroniki AA, Jackson D, Viechtbauer W, et al. Recommendations for quantifying the uncertainty in the summary intervention effect and estimating the between-study heterogeneity variance in random-effects meta-analysis. In: Chandler J, McKenzie J, Boutron I, et al., eds.: Cochrane Methods. Cochrane Database of Systematic Reviews 2015.
37. Hartung J, Knapp G. A refined method for the meta-analysis of controlled clinical trials with binary outcome. *Stat Med* 2001;20(24):3875-89. [published Online First: 2002/01/10]
38. Sidik K, Jonkman JN. A simple confidence interval for meta-analysis. *Stat Med* 2002;21(21):3153-9. doi: 10.1002/sim.1262 [published Online First: 2002/10/11]
39. RDevelopmentCoreTeam. R: A language and environment for statistical computing. Vienna, Austria: R Foundation for Statistical Computing, 2013.

For peer review only

40. Viechtbauer W. Conducting Meta-Analyses in R with the metafor Package. 2010;36(3):48.

41. DerSimonian R, Laird N. Meta-analysis in clinical trials. *Control Clin Trials* 1986;7(3):177-88.

42. Salanti G, Higgins JP, Ades AE, et al. Evaluation of networks of randomized trials. *Stat Methods Med Res* 2008;17(3):279-301. doi: 10.1177/0962280207080643 [published Online First: 2007/10/09]

43. Higgins JP, Whitehead A. Borrowing strength from external trials in a meta-analysis. *Stat Med* 1996;15(24):2733-49. doi: 10.1002/(SICI)1097-0258(19961230)15:24<2733::AID-SIM562>3.0.CO;2-0

44. Jackson D, White IR, Riley RD. Quantifying the impact of between-study heterogeneity in multivariate meta-analyses. *Stat Med* 2012;31(29):3805-20. doi: 10.1002/sim.5453 [published Online First: 2012/07/06]

45. Efthimiou O, Mavridis D, Debray TP, et al. Combining randomized and non-randomized evidence in network meta-analysis. *Stat Med* 2017;36(8):1210-26. doi: 10.1002/sim.7223 [published Online First: 2017/01/14]

46. Salanti G, Ades AE, Ioannidis JP. Graphical methods and numerical summaries for presenting results from multiple-treatment meta-analysis: an overview and tutorial. *J Clin Epidemiol* 2011;64(2):163-71. doi: 10.1016/j.jclinepi.2010.03.016 [published Online First: 2010/08/05]

47. Veroniki AA, Straus SE, Fyraridis A, et al. The rank-heat plot is a novel way to present the results from a network meta-analysis including multiple outcomes. *J Clin Epidemiol* 2016;76:193-9. doi: 10.1016/j.jclinepi.2016.02.016 [published Online First: 2016/03/05]

48. Rucker G, Schwarzer G. Ranking treatments in frequentist network meta-analysis works without resampling methods. *BMC medical research methodology* 2015;15:58. doi: 10.1186/s12874-015-0060-8 [published Online First: 2015/08/01]

49. Rücker G, Schwarzer G, Krahn U, et al. netmeta: Network Meta-Analysis using Frequentist Methods. R package version 0.9-8. 2018 [Available from: https://CRAN.R-project.org/package=netmeta.

50. Plummer M. rjags: Bayesian Graphical Models using MCMC. R package version 4-8 2018 [Available from: https://CRAN.R-project.org/package=rjags.

51. Salanti G. Indirect and mixed-treatment comparison, network, or multiple-treatments meta-analysis: many names, many benefits, many concerns for the next generation evidence synthesis tool. *Res Synth Methods* 2012;3(2):80-97. doi: 10.1002/jrsm.1037 [published Online First: 2012/06/11]

52. Jansen JP, Naci H. Is network meta-analysis as valid as standard pairwise meta-analysis? It all depends on the distribution of effect modifiers. *BMC Med* 2013;11:159. doi: 10.1186/1741-7015-11-159 [published Online First: 2013/07/04]

53. Konig J, Krahn U, Binder H. Visualizing the flow of evidence in network meta-analysis and characterizing mixed treatment comparisons. *Stat Med* 2013;32(30):5414-29. doi: 10.1002/sim.6001 [published Online First: 2013/10/15]

For peer review only

For peer review only - http://bmjopen.bmj.com/site/about/guidelines.xhtml

54. White IR, Barrett JK, Jackson D, et al. Consistency and inconsistency in network meta-analysis: model estimation using multivariate meta-regression. *Res Synth Methods* 2012;3(2):111-25. doi: 10.1002/jrsm.1045
55. Veroniki AA, Mavridis D, Higgins JP, et al. Characteristics of a loop of evidence that affect detection and estimation of inconsistency: a simulation study. *BMC medical research methodology* 2014;14:106. doi: 10.1186/1471-2288-14-106 [published Online First: 2014/09/23]
56. Song F, Clark A, Bachmann MO, et al. Simulation evaluation of statistical properties of methods for indirect and mixed treatment comparisons. *BMC medical research methodology* 2012;12:138. doi: 10.1186/1471-2288-12-138 [published Online First: 2012/09/14]
57. Turner RM, Davey J, Clarke MJ, et al. Predicting the extent of heterogeneity in meta-analysis, using empirical data from the Cochrane Database of Systematic Reviews. *Int J Epidemiol* 2012;41(3):818-27. doi: 10.1093/ije/dys041 [published Online First: 2012/03/31]
58. Chaimani A, Higgins JP, Mavridis D, et al. Graphical tools for network meta-analysis in STATA. *PLoS One* 2013;8(10):e76654. doi: 10.1371/journal.pone.0076654 [published Online First: 2013/10/08]
59. Chaimani A, Salanti G. Using network meta-analysis to evaluate the existence of small-study effects in a network of interventions. *Res Synth Methods* 2012;3(2):161-76. doi: 10.1002/jrsm.57 [published Online First: 2012/06/01]
60. Mavridis D, Efthimiou O, Leucht S, et al. Publication bias and small-study effects magnified effectiveness of antipsychotics but their relative ranking remained invariant. *J Clin Epidemiol* 2016;69:161-9. doi: 10.1016/j.jclinepi.2015.05.027 [published Online First: 2015/07/27]
61. Salanti G, Del Giovane C, Chaimani A, et al. Evaluating the quality of evidence from a network meta-analysis. *PLoS One* 2014;9(7):e99682. doi: 10.1371/journal.pone.0099682 [published Online First: 2014/07/06]

For peer review only

STATEMENTS

Declaration

The corresponding author had full access to all the data in the study and the final responsibility for the decision to submit for publication.
The senior author MK (the manuscript's guarantor) affirms that the manuscript is an honest, accurate, and transparent account of the study being reported and that no important aspects of the study have been omitted.

Author statement

The study was conceived and designed by MK, GS and EP. The protocol was drafted by AA, MK, AAV, OE, IK, GS and was revised critically for important intellectual content by all authors (AA, AAV, OE, IK, HN, SB, MP, PMH, PB, EP, GS, MK).

For peer review only - http://bmjopen.bmj.com/site/about/guidelines.xhtml

Acknowledgements
We thank the Jo's Trust for their assistance to involve public and patient representatives in this project.

Funding statement
This work is supported by National Institute for Health Research (NIHR) Research for Patient Benefits (P67307)(MK). MK is also supported by the British Society of Colposcopy Cervical Pathology Jordan/Singer Award (P47773) and the Imperial College Healthcare Charity (P47907)(MK). AAV is also supported by the European Union's Horizon 2020 (No 754936). None of the funders have any influence on the study design; in the collection, analysis, and interpretation of data; in the writing of the report; and in the decision to submit the article for publication.

Competing interests statement
We have read and understood BMJ policy on declaration of interests and declare that we have no conflict of interests. All authors have completed the ICMJE uniform disclosure form at www.icmje.org/coi_disclosure.pdf and declare: no support from any organisation for the submitted work; no financial relationships with any organisations that might have an interest in the submitted work in the previous three years; no other relationships or activities that could appear to have influenced the submitted work.
Dr Kyrgiou has received travel and conference expenses, honoraria and consultancy fees for commercial companies (Inovio, MSD etc); these activities are not related to the project.

Data sharing
No additional data available.

Copyright statement
"The Corresponding Author has the right to grant on behalf of all authors and does grant on behalf of all authors, a worldwide licence to the Publishers and its licensees in perpetuity, in all forms, formats and media (whether known now or created in the future), to i) publish, reproduce, distribute, display and store the Contribution, ii) translate the Contribution into other languages, create adaptations, reprints, include within collections and create summaries, extracts and/or, abstracts of the Contribution, iii) create any other derivative work(s) based on the Contribution, iv) to exploit all subsidiary rights in the Contribution, v) the inclusion of electronic links from the Contribution to third party material where-ever it may be located; and, vi) licence any third party to do any or all of the above."

For peer review only

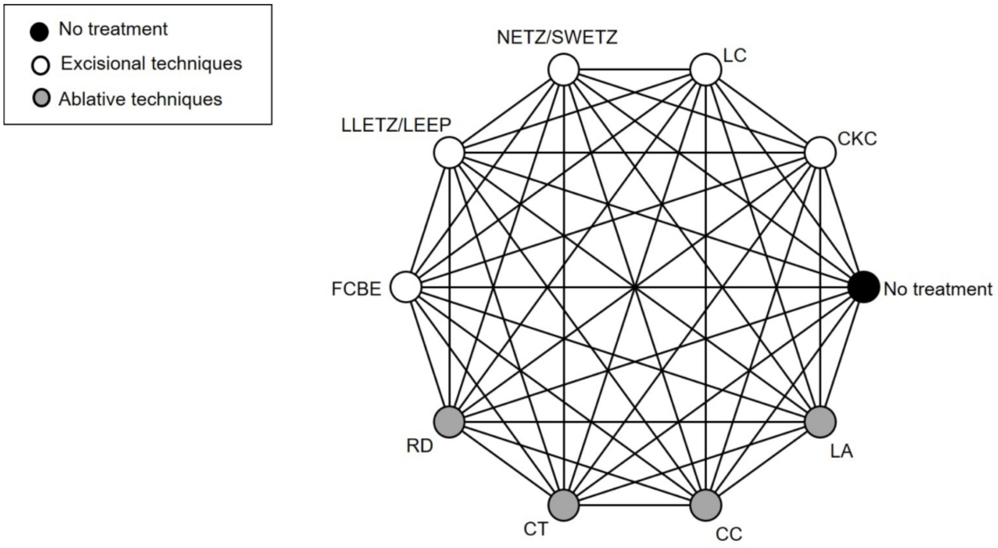

Network of possible pairwise comparisons between eligible treatment methods

203x112mm (300 x 300 DPI)

Supplementary File 1: Reporting checklist for protocol of a systematic review

Based on the PRISMA-P guidelines.

		Reporting Item	Page Number
Identification	#1a	Identify the report as a protocol of a systematic review	1
Update	#1b	If the protocol is for an update of a previous systematic review, identify as such	N/A (no previous NMA)
	#2	If registered, provide the name of the registry (such as PROSPERO) and registration number	2
Contact	#3a	Provide name, institutional affiliation, e-mail address of all protocol authors; provide physical mailing address of corresponding author	1
Contribution	#3b	Describe contributions of protocol authors and identify the guarantor of the review	15
	#4	If the protocol represents an amendment of a previously completed or published protocol, identify as such and list changes; otherwise, state plan for documenting important protocol amendments	4
Sources	#5a	Indicate sources of financial or other support for the review	15
Sponsor	#5b	Provide name for the review funder and / or sponsor	15
Role of sponsor or funder	#5c	Describe roles of funder(s), sponsor(s), and / or institution(s), if any, in developing the protocol	15
Rationale	#6	Describe the rationale for the review in the context of what is already known	3-4
Objectives	#7	Provide an explicit statement of the question(s) the review will address with reference to participants, interventions, comparators, and outcomes (PICO)	4
Eligibility criteria	#8	Specify the study characteristics (such as PICO, study design, setting, time frame) and report characteristics (such as years considered, language, publication status) to be used as criteria for eligibility for the review	4-5
Information sources	#9	Describe all intended information sources (such as electronic	5-6

For peer review only

		databases, contact with study authors, trial registers or other grey literature sources) with planned dates of coverage	
Search strategy	#10	Present draft of search strategy to be used for at least one electronic database, including planned limits, such that it could be repeated	5; Supplementary File 2
Study records - data management	#11a	Describe the mechanism(s) that will be used to manage records and data throughout the review	6
Study records - selection process	#11b	State the process that will be used for selecting studies (such as two independent reviewers) through each phase of the review (that is, screening, eligibility and inclusion in meta-analysis)	6
Study records - data collection process	#11c	Describe planned method of extracting data from reports (such as piloting forms, done independently, in duplicate), any processes for obtaining and confirming data from investigators	6
Data items	#12	List and define all variables for which data will be sought (such as PICO items, funding sources), any pre-planned data assumptions and simplifications	6
Outcomes and prioritization	#13	List and define all outcomes for which data will be sought, including prioritization of main and additional outcomes, with rationale	5
Risk of bias in individual studies	#14	Describe anticipated methods for assessing risk of bias of individual studies, including whether this will be done at the outcome or study level, or both; state how this information will be used in data synthesis	6-7
Data synthesis	#15a	Describe criteria under which study data will be quantitatively synthesised	7-8
	#15b	If data are appropriate for quantitative synthesis, describe planned summary measures, methods of handling data and methods of combining data from studies, including any planned exploration of consistency (such as I2, Kendall's τ)	7-9
	#15c	Describe any proposed additional analyses (such as sensitivity or subgroup analyses, meta-regression)	9
	#15d	If quantitative synthesis is not appropriate, describe the type of summary planned	N/A
Meta-bias(es)	#16	Specify any planned assessment of meta-bias(es) (such as	9

For peer review only

		publication bias across studies, selective reporting within studies)	
Confidence in cumulative evidence	#17	Describe how the strength of the body of evidence will be assessed (such as GRADE)	9

The PRISMA-P checklist is distributed under the terms of the Creative Commons Attribution License CC-BY 4.0. This checklist can be completed online using https://www.goodreports.org/, a tool made by the EQUATOR Network in collaboration with Penelope.ai

For peer review only

Supplementary File 2: Search algorithms

Medline Ovid

1 exp Uterine Cervical Neoplasms/
2 (cervi* and (cancer* or tumor* or tumour* or neoplas* or malignan* or carcinom*)).mp.
3 exp Cervical Intraepithelial Neoplasia/
4 CIN.mp.
5 (cervi* and (intraepithel* or epithel* or dysplasia or pre-cancer* or precancer*)).mp.
6 or/1-5
7 exp Conization/
8 (conisation or conization).mp.
9 exp Laser Therapy/
10 laser.mp.
11 exp Cryotherapy/
12 cryotherapy.mp.
13 cold coagulation.mp.
14 exp Diathermy/
15 diatherm*.mp.
16 cone biopsy.mp.
17 loop.mp.
18 LLETZ.mp.
19 LEEP.mp.
20 ablat*.mp.
21 excision*.mp.
22 transformation zone.mp.
23 (CKC or LA or LC or CC or RD or TZ).mp.
24 (conservative and (method* or treatment* or intervention* or management)).mp.
25 or/7-24
26 6 and 25
27 exp Premature Birth/
28 (preterm or premature).mp.
29 exp Infant, Low Birth Weight/
30 birth weight.mp.
31 Perinatal Mortality/
32 perinatal mortality.mp.
33 exp Intensive Care, Neonatal/
34 (neonatal and intensive care).mp.
35 exp Fertility/
36 fertil*.mp.
37 conception.mp.
38 exp Pregnancy/
39 pregnancy.mp.
40 gestation*.mp.
41 exp Abortion, Spontaneous/
42 miscarriage*.mp.
43 exp Cesarean Section/
44 (cesarean or caesarean).mp.
45 exp Obstetric Labor, Premature/
46 exp Labor, Obstetric/
47 (labor or labour).mp.

For peer review only

48 Fetal Membranes, Premature Rupture/
49 pPROM.mp.
50 or/27-49
51 26 and 50
key:
mp=title, original title, abstract, name of substance word, subject heading word

Embase Ovid
1 exp uterine cervix tumor/
2 (cervi* and (cancer* or tumor* or tumour* or neoplas* or malignan* or carcinom*)).mp.
3 uterine cervix carcinoma in situ/
4 CIN.mp.
5 (cervi* and (intraepithel* or epithel* or dysplasia or pre-cancer* or precancer*)).mp.
6 or/1-5
7 uterine cervix conization/
8 (conisation or conization).mp.
9 low level laser therapy/
10 laser.mp.
11 exp cryotherapy/
12 cryotherapy.mp.
13 cold coagulation.mp.
14 diathermy/
15 diatherm*.mp.
16 cone biopsy.mp.
17 loop.mp.
18 LLETZ.mp.
19 LEEP.mp.
20 ablat*.mp.
21 excision*.mp.
22 transformation zone.mp.
23 (CKC or LA or LC or CC or RD or TZ).mp.
24 (conservative and (method* or treatment* or intervention* or management)).mp.
25 or/7-24
26 6 and 25
27 prematurity/
28 (preterm or premature).mp.
29 exp low birth weight/
30 birth weight.mp.
31 perinatal mortality/
32 perinatal mortality.mp.
33 newborn intensive care/
34 (neonat* and intensive care).mp.
35 female fertility/
36 fertil*.mp.
37 conception/
38 conception.mp.
39 exp pregnancy/
40 pregnancy.mp.
41 gestation*.mp.
42 spontaneous abortion/

For peer review only

43 miscarriage*.mp.
44 cesarean section/
45 (cesarean or caesarean).mp.
46 premature labor/
47 (labor or labour).mp.
48 premature fetus membrane rupture/
49 pPROM.mp.
50 or/27-49
51 26 and 50
key:
mp=title, abstract, subject headings, heading word, drug trade name, original title, device manufacturer, drug manufacturer name

CENTRAL
#1 MeSH descriptor **Uterine Cervical Neoplasms** explode all trees
#2 cervi* and (cancer* or tumor* or tumour* or neoplas* or malignan* or carcinom*)
#3 MeSH descriptor **Cervical Intraepithelial Neoplasia** explode all trees
#4 CIN
#5 cervi* and (intraepithel* or epithel* or dysplasia or pre-cancer* or precancer*)
#6 (#1 OR #2 OR #3 OR #4 OR #5)
#7 MeSH descriptor **Conization** explode all trees
#8 conisation or conization
#9 MeSH descriptor **Laser Therapy** explode all trees
#10 laser
#11 MeSH descriptor **Cryotherapy** explode all trees
#12 cryotherapy
#13 cold coagulation
#14 MeSH descriptor **Diathermy** explode all trees
#15 diatherm*
#16 cone biopsy
#17 loop
#18 LLETZ
#19 LEEP
#20 ablat*
#21 excision*
#22 transformation zone
#23 CKC or LA or LC or CC or RD or TZ
#24 conservative and (method* or treatment* or intervention* or management)
#25 (#7 OR #8 OR #9 OR #10 OR #11 OR #12 OR #13 OR #14 OR #15 OR #16 OR #17 OR #18 OR #19 OR #20 OR #21 OR #22 OR #23 OR #24)
#26 (#6 AND #25)
#27 MeSH descriptor **Premature Birth** explode all trees
#28 preterm or premature
#29 MeSH descriptor **Infant, Low Birth Weight** explode all trees
#30 birth weight
#31 MeSH descriptor **Perinatal Mortality** explode all trees
#32 perinatal mortality
#33 MeSH descriptor **Intensive Care, Neonatal** explode all trees
#34 neonat* and (intensive care)
#35 MeSH descriptor **Fertility** explode all trees
#36 fertil*

For peer review only

#37 conception
#38 MeSH descriptor **Pregnancy** explode all trees
#39 pregnancy
#40 gestation*
#41 MeSH descriptor **Abortion, Spontaneous** explode all trees
#42 miscarriage*
#43 MeSH descriptor **Cesarean Section** explode all trees
#44 cesarean or caesarean
#45 MeSH descriptor **Obstetric Labor, Premature** explode all trees
#46 MeSH descriptor **Labor, Obstetric** explode all trees
#47 labor or labour
#48 MeSH descriptor **Fetal Membranes, Premature Rupture** explode all trees
#49 pPROM
#50 (#27 OR #28 OR #29 OR #30 OR #31 OR #32 OR #33 OR #34 OR #35 OR #36 OR #37 OR #38 OR #39 OR #40 OR #41 OR #42 OR #43 OR #44 OR #45 OR #46 OR #47 OR #48 OR #49)
#51 (#26 AND #50)

For peer review only
